# Ladder-like energy-relaying exciplex enables 100% internal quantum efficiency of white TADF-based diodes in a single emissive layer

**DOI:** 10.1038/s41467-021-23941-z

**Published:** 2021-06-15

**Authors:** Chunmiao Han, Ruiming Du, Hui Xu, Sanyang Han, Peng Ma, Jinkun Bian, Chunbo Duan, Ying Wei, Mingzhi Sun, Xiaogang Liu, Wei Huang

**Affiliations:** 1 Key Laboratory of Functional Inorganic Material Chemistry (Ministry of Education) & School of Chemistry and Material Science, Heilongjiang University, Harbin, PR China; 2 Department of Chemistry, National University of Singapore, Singapore, Singapore; 3 Key Laboratory of Flexible Electronics & Institute of Advanced Materials, Nanjing Tech University, Nanjing, China; 4 Frontiers Science Center for Flexible Electronics (FSCFE) & Shaanxi Institute of Flexible Electronics (SIFE), Northwestern Polytechnical University (NPU), Xi’an, China

**Keywords:** Electronic devices, Organic LEDs

## Abstract

Development of white organic light-emitting diodes based on purely thermally activated delayed fluorescence with a single-emissive-layer configuration has been a formidable challenge. Here, we report the rational design of a donor-acceptor energy-relaying exciplex and its utility in fabricating single-emissive-layer, thermally activated delayed fluorescence-based white organic light-emitting diodes that exhibit 100% internal quantum efficiency, 108.2 lm W^−1^ power efficiency, and 32.7% external quantum efficiency. This strategy enables thin-film fabrication of an 8 cm × 8 cm thermally activated delayed fluorescence white organic light-emitting diodes (10 inch^2^) prototype with 82.7 lm W^−1^ power efficiency and 25.0% external quantum efficiency. Introduction of a phosphine oxide-based acceptor with a steric group to the exciplex limits donor-acceptor triplet coupling, providing dual levels of high-lying and low-lying triplet energy. Transient spectroscopic characterizations confirm that a ladder-like energy relaying occurs from the high-lying triplet level of the exciplex to a blue emitter, then to the low-lying triplet level of the phosphine oxide acceptor, and ultimately to the yellow emitter. Our results demonstrate the broad applicability of energy relaying in multicomponent systems for exciton harvesting, providing opportunities for the development of third-generation white organic light-emitting diode light sources.

## Abstract (web summary)

Realizing efficient white organic light-emitting diodes (WOLEDs) with a single thermally-activated delayed fluorescence (TADF) emissive layer remains a challenge. Here, the authors design energy-relaying exciplex hosts with multi-triplet excited states for efficient single-emissive-layer TADF WOLEDs.

## Introduction

**Fig. 1:**
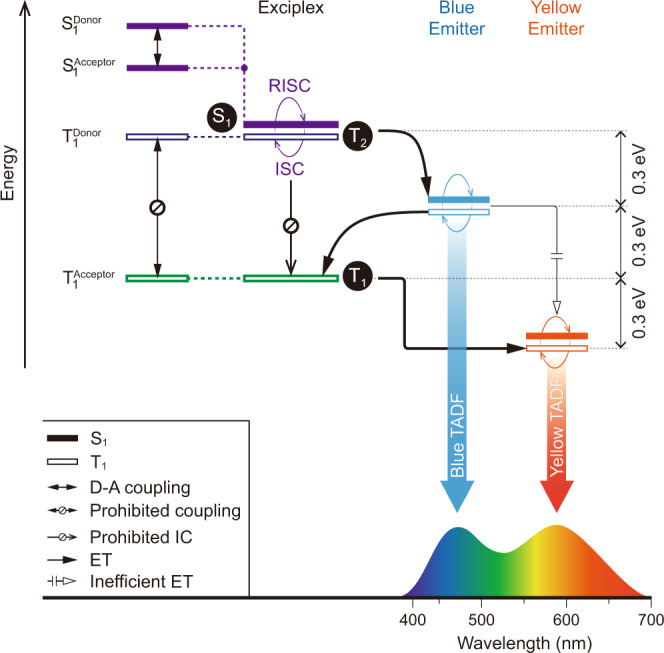
Proposed energy-relaying mechanism. Exciton allocation in single-emissive-layer, TADF WOLEDs comprising a D–A exciplex host, a blue emitter, and a yellow emitter. Inefficient energy transfer occurs from the exciplex host to the blue emitter and subsequently to the yellow emitter, due to a large energy gap between the blue and yellow emitters (~0.6 eV). The proposed exciplex, based on a high triplet donor and a low triplet acceptor, generates a large triplet gap between a high-lying **T**_**2**_ and a low-lying **T**_**1**_. This design enables efficient ladder-like (~0.3 eV ladder spacing) triplet energy transfer from **T**_**2**_ → blue emitter **→ T**_**1**_ → yellow emitter. ET, IC, ISC, and RISC refer to energy transfer, internal conversion, intersystem crossing, and reverse ISC, respectively.

Considerable effort has recently been devoted to developing high-efficiency, white organic light-emitting diodes (WOLEDs) with compact design and large-area processing capability^1–3^. Thermally activated delayed fluorescence (TADF), based on purely organic emitters, enables theoretical 100% internal quantum efficiency for both singlet and triplet exciton harvesting^4–6^. A single-emissive layer (EML) design, comprising blue/yellow emitters or red/green/blue emitters, can simplify WOLED device structure effectively and can meet the demands of large-scale production, quality control, and low cost^7–9^. However, competition in exciton confinement between various color emitters makes it challenging to control emission color and device efficiency synchronously (Fig. 1)^10,11^. As an additional constraint, charge-transfer excited states of TADF dyes are highly sensitive to host-dopant interactions^12–14^ and interfacial effects^15^. Indeed, there are few reports of high-efficiency, purely TADF-based WOLEDs, among which multiple emissive layers are required to modify exciton allocation by spatially separating two or three host-dopant systems of different emission color^16–18^.

High-efficiency WOLEDs with single-EML layout requires precise optimization of optical transition and energy transfer (ET) in a multi-emitter-doped host^19^. However, the narrow singlet-triplet splitting energy (ΔE_ST_) of TADF emitters limits energy-level regulation. The singlet-triplet energy gap between the blue and yellow TADF emitters, e.g., bis[4-(9,9-dimethyl-9,10-dihydroacridine)phenyl]sulfone (DMAC-DPS, S_1_/T_1_ ≈ 2.8 eV)^20^ and 2,3,5,6-tetrakis(3,6-di-*t*-butylcarbazol-9-yl)−1,4-dicyanobenzene (4CzTPNBu, S_1_/T_1_ ≈ 2.2 eV)^4^, is approximately 0.6 eV, leading to nonradiative deactivation of excitons during energy transfer^21–23^. Presently, the performance of single-EML, TADF-based WOLEDs is not comparable to that of their phosphorescent counterparts^24,25^. We reason that a host matrix with dual levels of high-lying and low-lying triplet energy, which match the energy levels of blue and yellow TADF emitters, may improve performance^23,26^. In contrast, single-molecule-based hosts cannot offer two triplet excited states, due to fast internal conversion to the lowest triplet state (T_1_). Therefore, an exciplex host based on a bimolecular donor–acceptor (D–A) system is likely to provide two different triplet states upon appropriate D–A coupling^27,28^. In principle, a common exciplex comprises a donor and an acceptor with comparable excited energy levels and strong D–A coupling, lowering S_1_ and T_1_ levels of the exciplex below those of the donor and the acceptor. In contrast, an exciplex design featuring a large triplet energy gap between the donor and the acceptor limits donor–acceptor triplet coupling (Fig. 1)^29^. This leads to dual triplet levels of the exciplex with an energy difference of 0.6 eV. Therefore, a facile triplet energy transfer process may occur, namely the S_1_/T_2_ of the exciplex host → the S_1_/T_1_ of blue emitter → the T_1_ of the exciplex host → the T_1_ of yellow emitter. As an added benefit, the exciplex host with near-zero ΔE_ST_ can provide additional triplet-singlet conversion and enhance singlet-exciton utilization in the blue-TADF emitter.

**Fig. 2:**
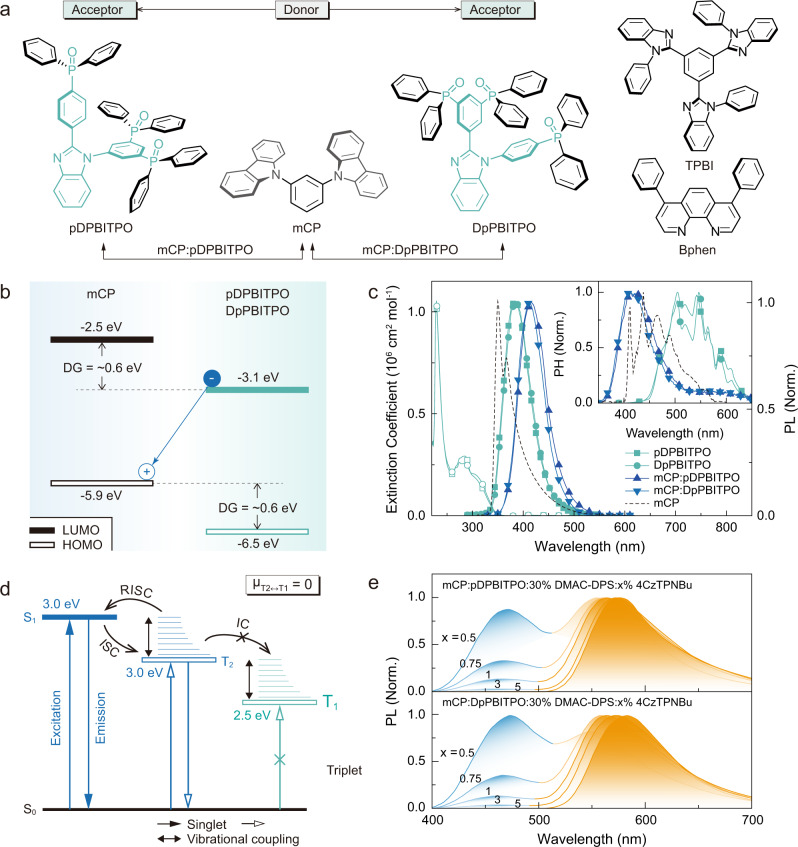
Structure and energy transition characteristics of the newly developed exciplexes. **a** Molecular structures of one donor and two acceptors used to form exciplexes mCP:pDPBITPO and mCP:DpPBITPO. Two conventional TPBI and BPhen acceptors were used in device fabrication for comparison. **b** Frontier molecular orbital (FMO) energy levels of mCP and PO acceptors. Energy gaps of the highest occupied (HOMO) and the lowest unoccupied molecular orbitals (LUMO) between mCP and PO acceptors reach to 0.6 eV, corresponding to the driving force of donor-acceptor electronic coupling (ΔG). **c** Electronic absorption of pDPBITPO and DpPBITPO (hollow dots) and steady-state photoluminescence (PL) and time-resolved phosphorescence (PH) spectra of mCP (dash lines), pDPBITPO, DpPBITPO, mCP:pDPBITPO and mCP:DpPBITPO. Phosphorescence spectra were recorded in the time range of 100-200 μs after excitation. **d** Jablonski energy level diagram of mCP:PO acceptors. The near-zero energy gap between the S_1_ and the mCP-induced T_2_ of the exciplex facilitates the RISC through electron and vibrational couplings. Separated T_2_ and T_1_ locations on mCP and PO acceptors restrain the T_2_ → T_1_ transition through IC. **e** Steady-state PL spectra of mCP:pDPBITPO and mCP:DpPBITPO films doped with DMAC-DPS and 4CzTPNBu at different ratios.

As a proof of concept, we designed and synthesized two exciplex hosts, mCP:pDPBITPO and mCP:DpPBITPO, with T_2_ and T_1_ energy levels of 3.0 and 2.5 eV, based on a high-triplet-energy donor, 1,3-bis(carbazol-9-yl)benzene (mCP, T_1_ = 3.0 eV) and low-triplet-energy phosphine oxide (PO) acceptors (5-(2-(4-(diphenylphosphoryl)phenyl)-benzimidazol-1-yl)−1,3-phenylene)bis(diphenylphosphine oxide) (pDPBITPO) and (5-(1-(4-(diphenylphosphoryl)phenyl)-benzoimidazol-2-yl)−1,3-phenylene)bis(diphenylphosphine oxide) (DpPBITPO) (T_1_ = 2.5 eV as the average of DMAC-DPS and 4CzTPNBu) (Fig. 2a). Our experimental results show that these two exciplexes promote energy transfer relaying in their DMAC-DPS and 4CzTPNBu-codoped films. A single-EML design based on mCP:pDPBITPO and mCP:DpPBITPO exciplex yielded TADF WOLEDs with a maximum external quantum efficiency (*η*_EQE_) of 32.7% and a maximum power efficiency of 108.2 lm W^−1^, comparable to that of fluorescent lamps.

## Results

### Exciplex host design

The key molecular design of pDPBITPO and DpPBITPO involves electron-accepting phenylbenzimidazole encapsulated by three diphenylphosphine oxide (DPPO) moieties to provide suitable frontier molecular orbital energy levels and to reduce intermolecular charge-transfer interactions with mCP^27^ (Fig. 2a). Furthermore, the single-crystal structure of DpPBITPO reveals a twisted configuration of the phenylbenzimidazole group due to steric hindrance of the DPPO moiety (Supplementary Fig. 1). This leads to improved triplet-state structural relaxation, reduced T_1_ energy level, and enlarged ΔE_ST_. Density functional theory (DFT) simulations show locally excited T_1_ states of mCP, pDPBITPO and DpPBITPO (Supplementary Fig. 2 and Supplementary Table 1). T_1_ energy gaps between the donor and the two acceptors reach ~0.8 eV, which is adequate to restrain D–A triplet exciton interactions. As a result of suppressed triplet D–A electronic coupling, high-lying triplet (T_2_) and T_1_ states of the exciplexes are localized at mCP and PO acceptors, respectively. Moreover, the energy gaps between the highest occupied (HOMO) and the lowest unoccupied molecular orbital (LUMO) energy levels of mCP and PO acceptors reach ~0.6 eV (Fig. 2b and Supplementary Figs. 3). This suggests strong driving forces of D–A charge transfer upon excitation, giving rise to charge-transfer S_1_ states. Effective spin-orbital coupling between the locally excited triplet state and the charge-transfer S_1_ state substantially facilitates reverse intersystem crossing (RISC), as evident in the cases of mCP:pDPBITPO and mCP:DpPBITPO (ref. ^30^). Moreover, PO acceptors are superior to conventional low-triplet-energy acceptors with exposed T_1_ states, such as TPBI (T_1_ = 2.7 eV) and BPhen (T_1_ = 2.6 eV), due to prevention of collision-induced quenching at the T_1_ state by peripheral DPPO groups.

Estimated by 0 → 0 transitions in emission spectra, the T_1_ energy levels (2.48 eV) of pDPBITPO and DpPBITPO match well with 4CzTPNBu (2.2 eV), while featuring triplet gaps that are 0.54 eV lower than mCP (T_1_ = 3.02 eV). Meanwhile, the S_1_ energy levels of the PO acceptors are 3.2 eV, similar to mCP (S_1_ = 3.5 eV). The deep-blue emissions from co-evaporated mCP:PO acceptor films exhibit a bathochromic shift of 30 nm, corresponding to Gibbs free energy for charge separation beyond 0.2 eV, indicating exciplex formation through D–A electronic coupling (Fig. 2c; Supplementary Figs. 4 and 5; Supplementary Table 2) (ref. ^29^). The D–A vibrational coupling leads to structureless phosphorescence spectra of mCP:pDPBITPO and mCP:DpPBITPO. The main peaks at 415 nm correspond to the 0 → 0 triplet transition of mCP, namely the mCP-centered T_2_ → S_0_ transitions. Therefore, the T_2_ levels of the exciplexes (~3.0 eV) support efficient energy transfer to DMAC-DPS. Furthermore, the tails of the phosphorescence spectra from 500 to 650 nm overlap with those of the PO acceptors, which can be ascribed to either the T_1_ → S_0_ transitions of the exciplexes or to triplet leakage to the T_1_ state of acceptors^29^. Compared to T_1_ states located at the PO acceptor, mCP-induced T_2_ states are closer to S_1_ states, supporting RISC transitions. In the case of weak D–A triplet exciton interactions and mCP-centralized HOMOs, these T_2_ states dominate the triplet population. Furthermore, D–A vibrational coupling restrains vibrational perturbation of potential energy surfaces and further alleviates internal conversion from the T_2_ to T_1_ states (Fig. 2d), closely resembling rigid, intramolecular charge-transfer systems^31^. Despite the decreased photoluminescence quantum yields (*ϕ*_PL_) of the exciplexes (<15%) (Supplementary Table 2), the PO acceptor-induced T_1_ states can serve as intermediate states to promote triplet energy transfer to 4CzTPNBu.

### Photophysical investigation on the energy-relaying process

The DMAC-DPS (30 wt%) and 4CzTPNBu (5 wt%) singly doped sky-blue and yellow exciplex films (100 nm) showed ~30% and ~65% increased *ϕ*_PL_, respectively (Fig. 2e; Supplementary Table 2). These values are still far below the intrinsic values of the dopants (~95%), due to either triplet leakage to the T_1_ of the PO acceptors (for DMAC-DPS) or the large energy gap-induced inefficient energy transfer (for 4CzTPNBu). For the doubly doped films of mCP:PO acceptor:30% DMAC-DPS:x% 4CzTPNBu (x = 0.5-5), they all achieved *ϕ*_PL_ values above 90%, approximately the sum of the singly doped films.

**Fig. 3:**
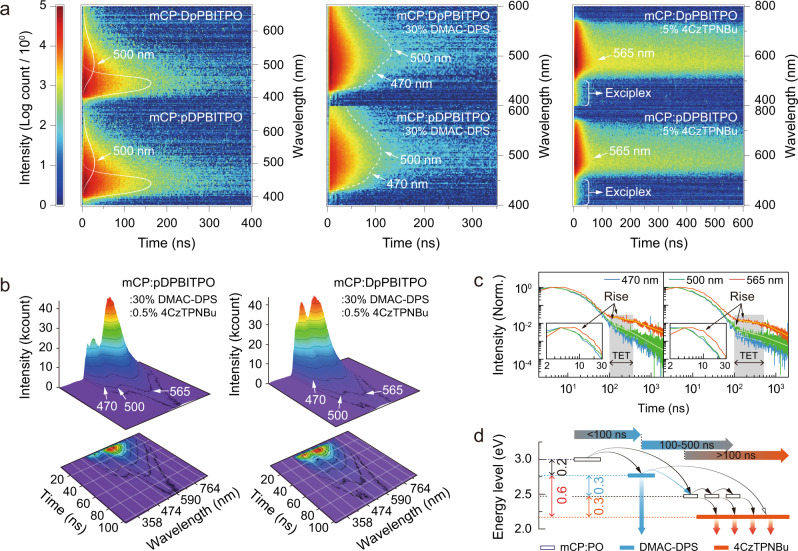
Transient emission properties of the exciplexes and their TADF dye-doped films. **a** Time-dependent emission contours of mCP:pDPBITPO and mCP:DpPBITPO films, 30% DMAC-DPS-doped exciplex films and 4CzTPNBu-doped (5%) exciplex films, measured in the range of 350–600 nm. **b** Time-dependent emission intensity profiles of mCP:pDPBITPO and mCP:DpPBITPO films doped with 30 wt% DMAC-DPS and 0.5 wt% 4CzTPNBu within 120 ns after excitation. **c** Time-decay curves of mCP:pDPBITPO (left) and mCP:DpPBITPO (right) films doped with 30 wt.% DMAC-DPS and 0.5 wt% 4CzTPNBu. TET refers to a triplet energy transfer from DMAC-DPS to phosphine oxide (PO) acceptors. **d** Proposed time-dependent energy transfer processes in dually-doped exciplex films, showing a ladder-like energy relaying enabled by the low-lying T_1_ energy level of the PO acceptor.

The weakly populated bands at 500 nm, observed in the time-resolved contours of mCP:PO acceptor exciplexes due to the phosphorescence from the PO acceptors, could be dramatically enhanced upon doping of 30% DMAC-DPS (Fig. 3a). The triplet energy was transferred from DMAC-DPS to PO acceptors at about 70 ns, followed by the appearance of dominant phosphorescence bands at 150 ns. For mCP:PO acceptor:5% 4CzTPNBu, we only observed blue bands from the exciplexes within 50 ns. The absence of phosphorescence bands indicates that PO acceptors do not participate in triplet energy transfer to 4CzTPNBu due to forbidden T_2_ → T_1_ transitions of the exciplexes. The time-resolved emission spectra (TRES) of mCP:PO acceptor:30% DMAC-DPS:0.5% 4CzTPNBu films were measured to examine the exact energy transfer process (Fig. 3b). In the time range of 100–500 ns, along with the decrease of the bands assigned to DMAC-DPS (~470 nm), the bands that peaked at ~500 nm gradually became dominant, which could be attributed to phosphorescence from the PO acceptors. After the coexistence of the phosphorescent and yellow bands during this time range, the yellow bands remained while the phosphorescence bands vanished. Furthermore, despite the low 4CzTPNBu concentration (0.5%), the DF lifetimes (τ_DF_) of blue bands of DMAC-DPS singly doped films (x = 0) decreased from 1 to 0.5 μs while the τ_DF_ values of yellow bands decreased to 0.7 μs (Fig. 3c; Supplementary Table 2). With a large distance between the dopants, energy transfer depends largely on the intermediate T_1_ energy levels of the PO acceptors for exciton migration from blue to yellow dopants.

Apart from the ultrafast intensity rise (6 ns) in yellow bands (insets in Fig. 3c), we recorded appreciable and nearly synchronous rises in both phosphorescent and yellow bands within 100–500 ns. DMAC-DPS with high-lying excited states supports additional energy transfer to the T_1_ state of the PO acceptor and then 4CzTPNBu. These synchronous rises further suggest coordination of the exciplex and DMAC-DPS in facilitating energy transfer to 4CzTPNBu. Therefore, upon excitation, a fast, singlet energy transfer occurs, and the triplet energy can be immediately transferred from the exciplexes to DMAC-DPS and then to the T_1_ energy levels of the PO acceptors within 100–500 ns (Fig. 3d). Almost instantly, the PO acceptors provide the triplet-exciton migration and energy transfer to 4CzTPNBu. As a result, energy harvesting is improved by energy relaying that occurs between blue and yellow dyes, namely mCP:PO acceptor → DMAC-DPS → PO acceptor → 4CzTPNBu.

### WOLED performance

**Fig. 4:**
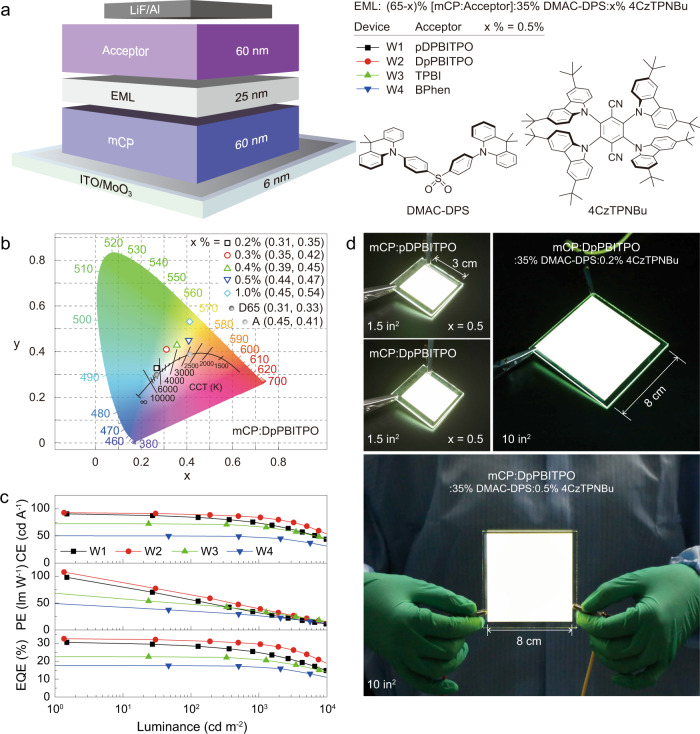
Electroluminescence characterizations of single-emissive-layer TADF WOLEDs comprising ladder-like energy-relaying exciplexes. **a** An illustration showing device configuration, the emissive layer components of mCP:pDPBITPO- and mCP:DpPBITPO-hosted white TADF devices, and molecular structures of the doped dye emitters. **b** Commission International de I’Eclairage (CIE) coordinates of mCP:DpPBITPO-hosted TADF WOLEDs, fabricated with mCP:DpPBITPO:35% DMAC-DPS:x%4CzTPNBu. Their chromaticity is comparable to that of a standard daylight source (Illuminant D65) or an incandescent light (Illuminant A). **c** Efficiency *vs*. luminance measurements of the devices based on 35% DMAC-DPS and 0.5% 4CzTPNBu codoped mCP:pDPBITPO (W1), mCP:DpPBITPO (W2), mCP:TPBI (W3) and mCP:BPhen (W4). **d** Photographs of mCP:pDPBITPO- and mCP:DpPBITPO-hosted WOLED prototypes with lateral dimensions of 3 cm **×** 3 cm (1.5 inch^2^) and 8 cm **×** 8 cm (10 inch^2^).

Based on the high *ϕ*_PL_ values of dual-doped exciplex films and the excellent electrical performance of the PO acceptors and their exciplexes (Supplementary Fig. 3; Supplementary Tables 1 and 2), we further constructed two trilayer TADF-based WOLEDs (W1 and W2) with doubly doped, single-emissive layers (Fig. 4a; Supplementary Fig. 6). Through concentration tuning of 4CzTPNBu, we achieved color modulation from pure white to warm white with a color rendering index beyond 80 and with high chromatic stability (Fig. 4b; Supplementary Figs. 7 and 8; Supplementary Table 3). Commission Internationale de l’Eclairage (CIE) coordinates and correlated color temperatures were (0.31, 0.35)/6582 K for the pure white device and (0.44, 0.47)/3474 K for the warm white device. Their chromatic purity compared well with a standard daylight source (Illuminant D65) and an incandescent light (Illuminant A).

For the mCP:pDPBITPO-based device W1, we achieved maximum efficiencies of 98.4 lm W^−1^ and 30.6%. mCP:DpPBITPO-derived W2 generated a power efficiency of 108.2 lm W^−1^, an external quantum efficiency of 32.7% and a device lifetime of 233 h at half-initial luminance (Fig. 4c; Supplementary Figs. 9–11). Device degradation is mainly due to photo- and electro-decomposition of DMAC-DPS (Supplementary Figs. 12 and 13). So, using another more stable blue TADF emitter 4,5-bis(carbazol-9-yl)−1,2-dicyanobenzene (2CzPN) instead of DMAC-DPS (Supplementary Fig. 14), device lifetime can be doubled (Supplementary Fig. 11). Based on a 30% out-coupling ratio of an indium tin oxide glass, we derived 100% internal quantum efficiencies (*η*_IQE_) for both W1 and W2. Large-size WOLEDs are hard to fabricate due to stringent requirements on defect control and device homogeneity. We next fabricated TADF WOLED prototypes with lateral dimensions of 8 cm **×** 8 cm and efficiencies of 82.7 lm W^−1^ and 25.0% (Fig. 4d, Supplementary Fig. 15). Our approach is applicable to conventional acceptors. For example, devices W3 and W4, based on two commercial TPBI and BPhen acceptors, achieved maximum efficiencies of 71.3 lm W^−1^ and 22.6% (Fig. 4c, Supplementary Table 3). In addition, our strategy enabled phosphorescent and hyperfluorescent WOLEDs with respective external quantum efficiencies of 32.4% and 13.7% (Supplementary Fig. 16).

It is interesting that the maximum η_EQE_ values of W1 and W2 are larger than the sum of the corresponding singly doped blue and yellow devices (Supplementary Figs. 17 and 18). We further doped 30% DMAC-DPS in the emissive layer of the yellow device (Supplementary Fig. 19). Incorporation of DMAC-DPS dramatically improved the efficiencies by ~40%. Furthermore, we fabricated an mCP:DpPBITPO-based WOLED with dual emissive layers, in which DMAC-DPS and 4CzTPNBu were spatially separated to interrupt triplet energy transfer from DMAC-DPS to DpPBITPO to 4CzTPNBu. This reduced maximum efficiencies to 9.3%, due to triplet leakage to the T_1_ levels of DpPBITPO in the blue-emissive layer and nonradiative energy loss in the yellow-emissive layer (Supplementary Fig. 20). Taken together, the exciplexes foster energy relaying between the blue and yellow dopants for high-efficiency white electroluminescence.

## Discussion

Our work suggests a general approach to realizing single-emissive-layer TADF WOLEDs with fluorescent lamp efficiency. This strategy, based on exciplex hosts with energy levels simultaneously matching those of blue and yellow dopants, enables a ladder-like, high-efficiency energy relaying. Implementation of these exciplex hosts leads to 100% exciton harvesting. Furthermore, the realization of purely TADF-based WOLEDs in a simple trilayer device provides a sustainable, cost-effective approach to mitigating fabrication complexity. Ultimately, our molecular design may offer fundamental insight into energy transfer in multicomponent white-emitting systems and add value to the construction of next-generation lighting sources.

## Methods

### Preparation of PO acceptors

We designed and synthesized two phosphine oxide acceptors (pDPBITPO and DpPBITPO). These acceptors were fully characterized by ^1^H NMR, 13 C NMR, 31 P NMR, MS, elemental analysis, and single-crystal (if available) X-ray diffraction analysis (Supplementary Figs 21–26).

### General procedure of the cyclization reaction

In an argon atmosphere, aromatic aldehyde (1 mmol) and arylamine (1 mmol) were dissolved in DMF (5 mL). The resulting solution was heated to 80 °C. A saturated aqueous Na_2_S_2_O_5_ solution (1 mL) was then added to the mixture and stirred for 20 h at 80 °C. After cooling to room temperature, the mixture was poured into water. The precipitate was filtered as a crude product and further recrystallized with methanol to afford phenylbenzimidazole derivatives.

#### 2-(4-Bromophenyl)-1-(3,5-dibromophenyl)-1H-benzo[d]imidazole (pDPBITBr)

prepared from 4-bromobenzaldehyde and *N*-(3,5-dibromophenyl)benzene-1,2-diamine to afford white powder in 73% yield. ^1^H NMR (TMS, CDCl_3_, 400 MHz): 7.880 (d, *J* = 8.0 Hz, 1H), 7.809 (m, 1H), 7.532 (d, *J* = 8.4 Hz, 2H), 7.454 (d, *J* = 8.8 Hz, 4H), 7.388 (t, *J* = 7.2 Hz, 1H), 7.36 (t, *J* = 7.2 Hz, 1H), 7.259 ppm (d, *J* = 8.0 Hz, 1H); ^13^C NMR (TMS, CDCl_3_, 100 MHz): 149.900, 141.878, 137.911, 135.705, 133.655, 130.916, 129.751, 128.253, 127.122, 123.642, 123.131, 122.843, 122.730, 119.244, 109.057 ppm; HRMS (MALDI-TOF) m/z: [M + H^+^] 506.875; elemental analysis (%) for C_19_H_11_Br_3_N_2_: C 45.01, H 2.19, N 5.53; found: C 45.03, H 2.18, N 5.55.

#### 1-(4-Bromophenyl)-2-(3,5-dibromophenyl)-1H-benzo[d]imidazole (DpPBITBr)

prepared from 3,5-dibromobenzaldehyde and *N*-(4-bromophenyl)benzene-1,2-diamine to afford white powder in 71% yield. ^1^H NMR (TMS, CDCl_3_, 400 MHz): 7.889 (d, *J* = 7.6 Hz, 1H), 7.712 (d, *J* = 8.4 Hz, 2H), 7.676 (t, *J* = 1.6 Hz, 1H), 7.644 (d, *J* = 1.6 Hz, 2H), 7.395 (td, *J* = 7.6 Hz, 1.2 Hz, 1H), 7.337 (td, *J* = 7.6 Hz, 1.2 Hz, 1H), 7.244-7.175 ppm (m, 3H); ^13^C NMR (TMS, CDCl_3_, 100 MHz): 147.946, 141.754, 135.966, 134.279, 134.031, 132.437, 131.971, 129.887, 127.821, 123.335, 122.678, 122.055, 121.942, 119.321, 109.379 ppm; HRMS (MALDI-TOF) m/z: [M + H^+^] 506.875; elemental analysis (%) for C_19_H_11_Br_3_N_2_: C 45.01, H 2.19, N 5.53; found: C 45.02, H 2.19, N 5.56.

### General procedure of phosphorylation

In an argon atmosphere, bromide (1 mmol), NaOAc (3.3 mmol), Pd(OAc)_2_ (0.15 mmol) and diphenylphosphine (3.3 mmol) were dissolved in DMF (10 mL). The mixture was heated to 130 ^o^C and kept at this temperature for 36 h under stirring. After cooling to room temperature, the reaction was quenched with water (20 mL). The solution was then extracted with CH_2_Cl_2_ (3 × 20 mL). The organic layers were combined and dried with anhydrous sodium sulfate. The solvent was removed *in vacuo* to afford phosphine precursors. Then, the phosphines were dissolved in CH_2_Cl_2_ (10 mL) and H_2_O_2_ (30%, 3 mL) was added to the CH_2_Cl_2_ solution dropwise at 0 °C. The mixture was stirred for 2 h, followed by extraction with CH_2_Cl_2_ (3 × 10 mL). The organic layers were combined and dried with anhydrous sodium sulfate. The solvent was removed *in vacuo*, and the residue was purified by flash column chromatography to afford phosphine oxide derivatives.

#### (5-(2-(4-(Diphenylphosphoryl)phenyl)-1H-benzo[d]imidazol-1-yl)-1,3-phenylene)bis(diphenylphosphine oxide) (pDPBITPO)

prepared from pDPBITBr to afford white powder in 67% yield. ^1^H NMR (TMS, CDCl_3_, 400 MHz): 7.878-7.757 (m, 4H), 7.723 (dd, *J*_1_ = 8.0 Hz, *J*_2_ = 11.6 Hz, 2H), 7.653 (dd, *J*_1_ = 8.0 Hz, *J*_2_ = 11.6 Hz, 4H), 7.584-7.518 (m, 5H), 7.518-7.418 (m, 15H), 7.418-7.343 (m, 8H), 7.343-7.291 (m, 1H), 7.258 (t, *J* = 7.6 Hz, 1H), 7.065 ppm (d, *J* = 8.0 Hz, 1H); ^13^C NMR (TMS, CDCl_3_, 100 MHz): 149.765, 141.950, 136.643, 136.531, 136.472, 136.338, 135.668, 135.555, 135.386, 134.038, 133.941, 133.847, 133.802, 132.789, 132.618, 132.535, 131.878, 131.850, 131.581, 131.274, 131.226, 131.199, 131.178, 131.053, 130.953, 130.763, 130.711, 130.660, 130.548, 130.200, 129.148, 128.474, 128.355, 127.924, 127.859, 127.796, 127.754, 127.632, 123.410, 122.848, 119.402, 109.656 ppm; ^31^P NMR (TMS, CDCl_3_, 160 MHz): 28.195, 27.637 ppm; HRMS (MALDI-TOF) m/z: [M + H^+^] 871.323; elemental analysis (%) for C_55_H_41_N_2_O_3_P_3_: C 75.86, H 4.75, N 3.22; found: C 75.88, H 4.76, N 3.25.

#### (5-(1-(4-(Diphenylphosphoryl)phenyl)-1H-benzo[d]imidazol-2-yl)-1,3-phenylene)bis(diphenylphosphine oxide) (DpPBITPO)

prepared from DpPBITBr to afford white powder in 69% yield. ^1^H NMR (TMS, CDCl_3_, 400 MHz): 8.109 (d, *J* = 12.4 Hz, 2H), 7.901 (t, *J* = 11.2 Hz, 1H), 7.841–7.744 (m, 3H), 7.730 (dd, *J*_1_ = 7.6 Hz, *J*_2_ = 12.0 Hz, 4H), 7.577 (m, 2H), 7.539–7.430 (m, 16H), 7.421–7.302 (m, 9H), 7.302–7.241 (m, 3H), 7.191 ppm (d, *J* = 8.0 Hz, 1H); ^13^C NMR (TMS, CDCl_3_, 100 MHz): 148.973, 141.925, 138.369, 138.339, 135.757, 135.039, 134.937, 134.908, 134.829, 134.798, 134.261, 134.156, 133.272, 133.187, 133.161, 133.122, 133.057, 132.952, 132.264, 131.309 131.234, 131.146, 131.046, 130.917, 130.866, 130.814, 130.702, 130.188, 129.656, 127.884, 127.794, 127.762, 127.734, 127.668, 126.195, 126.072, 123.349, 122.737, 119.345, 109.388 ppm; ^31^P NMR (TMS, CDCl_3_, 160 MHz): 28.437, 27.966 ppm; HRMS (MALDI-TOF) m/z: [M + H^+^] 871.351; elemental analysis (%) for C_55_H_41_N_2_O_3_P_3_: C 75.86, H 4.75, N 3.22; found: C 75.88, H 4.76, N 3.25.

### DFT calculations

DFT computations were performed with different parameters for structural optimization and vibrational analysis. The ground state configuration of DpPBITPO was established according to single-crystal data. Ground, singlet, and triplet states in vacuum were optimized by restricted and unrestricted formalisms of Beck’s three-parameter hybrid exchange functional^32^ and Lee, and Yang and Parr correlation functional^33^ B3LYP/6-31 G(d,p), respectively. Fully optimized stationary points were further characterized by harmonic vibrational frequency analysis to ensure a real, local minimum without imaginary vibrational frequency. Total energies were also corrected using zero-point energy for both the ground and triplet states. Contours were visualized using Gaussview 5.0. All computations were performed using a Gaussian 09 software package.

### Device fabrication and testing

Before loading into a deposition chamber, the ITO substrate was cleaned with detergent and deionized water, dried in an oven at 120 °C for 4 h, and treated with oxygen plasma for 3 min. Devices were fabricated by evaporating organic layers at a rate of 0.1–0.2 nm s^−1^ onto the ITO substrate sequentially at a pressure below 4 × 10^−4^ Pa. Onto the electron-transporting layer, a layer of LiF with 1-nm thickness was deposited at a rate of 0.1 nm s^−1^ to improve electron injection. Finally, a 100-nm layer of Al was deposited at a rate of 0.6 nm s^−1^ as the cathode. The emission area of the devices was 0.09 cm^2^, as determined by the overlapped area of the anode and the cathode. After fabrication, devices were immediately transferred to a glove box for encapsulation with glass coverslips using epoxy glue. EL spectra and CIE coordinates were measured using a PR655 spectrum colorimeter. Current–density–voltage and brightness-voltage curves of the devices were measured using a Keithley 4200 source meter and a calibrated silicon photodiode. All measurements were carried out at room temperature under ambient conditions. For each structure, four devices were fabricated in parallel to confirm performance repeatability. The data reported herein were those closest to the average results.

### Photophysical measurement

Steady-state emission spectra were measured using an Edinburgh FPLS 920 fluorescence spectrophotometer. TADF dye-doped films (100 nm) were prepared by vacuum evaporation for optical analysis. Photoluminescence quantum yields (PLQY, *ϕ*_PL_) of these films were measured through a Labsphere 1-M-2 (*ϕ* = 6”) integrating sphere coated with Benflect having efficient light reflection from 200 to 1600 nm, which was integrated with FPLS 920. The absolute *ϕ*_PL_ determination of the sample was performed with two spectral (emission) scans, with the emission monochromator scanning over the Rayleigh scattered light from the sample and a blank substrate. The first spectrum recorded the scattered light and the sample emission, and the second spectrum recorded the scattered light of the Benflect coating. Integration and subtraction of the scattered light in the two spectra are equal to the number of photons absorbed by the samples (N_a_), while integration of the sample emission is equal to the number of photons emitted (N_e_). Then, absolute *ϕ*_PL_ can be estimated according to the equation of *ϕ*_PL_ = N_e_/N_a_. Spectral correction (emission arm) was applied to raw data after background subtraction, and from these spectrally corrected curves, the quantum yield was calculated using an F900 software wizard.

Time-resolved photoluminescence measurements with nanosecond time resolution were acquired with a gated, intensified CCD camera system (Andor iStar DH740 CCI-010) connected to a grating spectrometer (Andor SR303i) at room temperature. Excitation (wavelength: 350 nm) was performed with femtosecond laser pulses, generated by introducing 1 kHz pulses (pulse length: 80 fs) from a central Ti:sapphire amplifier system (Spectra-Physics Solstice) to a TOPAS optical parametric amplifier (Light Conversion). Time-resolved photoluminescence of the sample was obtained by stepping the intensified CCD gate delay relative to the pump pulse. The gate width was 2 ns.

## Supplementary information

Supplementary Information

## Data Availability

Data that support the findings of this study are available from the corresponding authors upon request. The X-ray crystallographic coordinates for the DpPBITPO structure reported in this study have been deposited at the Cambridge Crystallographic Data Centre (CCDC), under deposition numbers 2077630. These data can be obtained free of charge from The Cambridge Crystallographic Data Centre via www.ccdc.cam.ac.uk/data_request/cif.
