# Supplementary material for: Ladder-like energy-relaying exciplex enables 100% internal quantum efficiency of white TADF-based diodes in a single emissive layer
Source: Nat Commun. 2021 Jun 15;12:3640. doi: 10.1038/s41467-021-23941-z (PMC8206214; doi:10.1038/s41467-021-23941-z)
Supplement: Supplementary file 1 — Supplementary Information [file 41467_2021_23941_MOESM1_ESM.pdf]

Supplementary Information

Ladder-like energy relaying exciplex enables 100% internal quantum efficiency of white TADF-based diodes in a single emissive layer

Chunmiao Han[1], Ruiming Du[1], Hui Xu[1]*, Sanyang Han[2], Peng Ma[1], Jinkun Bian[1], Chunbo Duan[1], Ying Wei[1], Mingzhi Sun[1], Xiaogang Liu[2]*& Wei Huang[3,4]*

[1]Key Laboratory of Functional Inorganic Material Chemistry (Ministry of Education) & School of Chemistry and Material Science, Heilongjiang University, 74 Xuefu Road, Harbin 150080, P. R. China. [2]Department of Chemistry, National University of Singapore, Singapore 117543, Singapore. [3]Key Laboratory of Flexible Electronics & Institute of Advanced Materials, Nanjing Tech University, Nanjing 211816, China. [4]Frontiers Science Center for Flexible Electronics (FSCFE) & Shaanxi Institute of Flexible Electronics (SIFE), Northwestern Polytechnical University (NPU), 127 West Youyi Road, Xi'an 710072, China.

Materials and Instruments. ^{1}H NMR spectra were recorded using a Varian Mercury plus 400NB spectrometer relative to tetramethylsilane (TMS) as the internal standard. Molecular masses were determined by a MALDI-TOF-MS. Elemental analyses were performed on a Vario EL III elemental analyzer. The crystal suitable for single-crystal XRD analysis was obtained by slowly diffusing hexane into a dichloromethane solution of ***Dp*PBITPO** at room temperature. All diffraction data were collected at 295 K on a Rigaku Xcalibur E diffractometer with graphite monochromatized Mo K_α (λ = 0.71073 Å) radiation in ω scan mode. All structures were solved by direct method and difference Fourier syntheses. Non-hydrogen atoms were refined by full-matrix least-squares techniques on F2 with anisotropic thermal parameters. The hydrogen atoms attached to carbons were placed in calculated positions with C−H = 0.93 Å and U(H) = 1.2Ueq(C) in the riding model approximation. All calculations were carried out with the SHELXL97 program. Absorption spectra of the target compound were measured using a SHIMADZU UV-3150 spectrophotometer. Thermogravimetric analysis (TGA) and differential scanning calorimetry (DSC) were performed on Shimadzu DSC-60A and DTG-60A thermal analyzers under a nitrogen atmosphere at 10 ºC min^{-1} heating rate. The morphological characteristics of the vacuum-evaporated films were measured with an atom force microscope (AFM) Agilent 5100 under tapping mode. Cyclic voltammetric (CV) studies

were conducted using an Eco Chemie B. V. AUTOLAB potentiostat in a typical three-electrode cell with a glassy carbon working electrode, a platinum wire counter electrode, and a silver/silver chloride (Ag/AgCl) reference electrode. All electrochemical experiments were carried out under a nitrogen atmosphere at room temperature in dichloromethane.

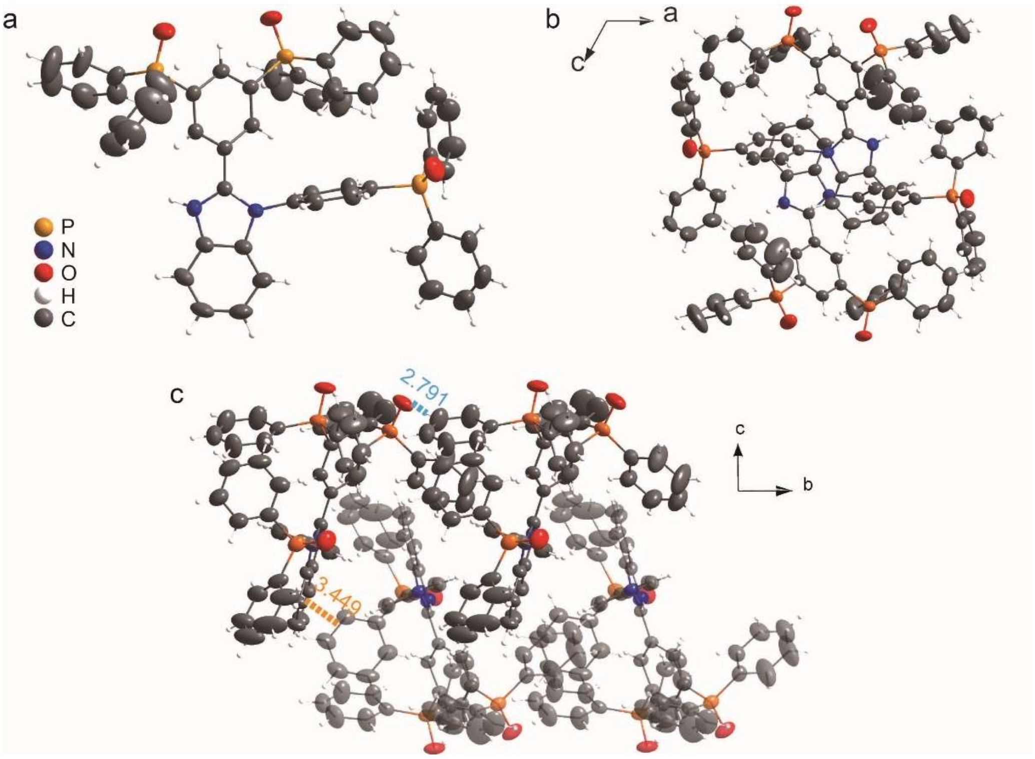

Supplementary Fig. 1 Single-crystal data of DpPBITPO. **a,** Single-crystal structure with C, N, O and P atoms in the ellipsoid model shown at a 50% level and H atoms in a ball-stick mode for clarity. Selected bond lengths (Å) and angles (°): P1-O1 1.474(9), P2-O2 1.479(2), P3-O3 1.472(2), N1-C1 1.379(6), N2-C1 1.303(6), the dihedral angle between 1-phenyl and imidazole ring = 29.553°. **b-c,** Perspective views of packing diagrams along *b* (**b**) and *a* (**c**) axes. Two opposite molecular orientations form alternant 2D networks along *a* axis. Between two adjacent molecules in the same 2D network, P=O---H hydrogen bondings have a distance of 2.791(3) Å. Between different 2D networks, centroid-to-edge π-π interactions have a distance of 3.449(3) Å. These abundant intermolecular interactions facilitate charge hopping for carrier injection and transportation.

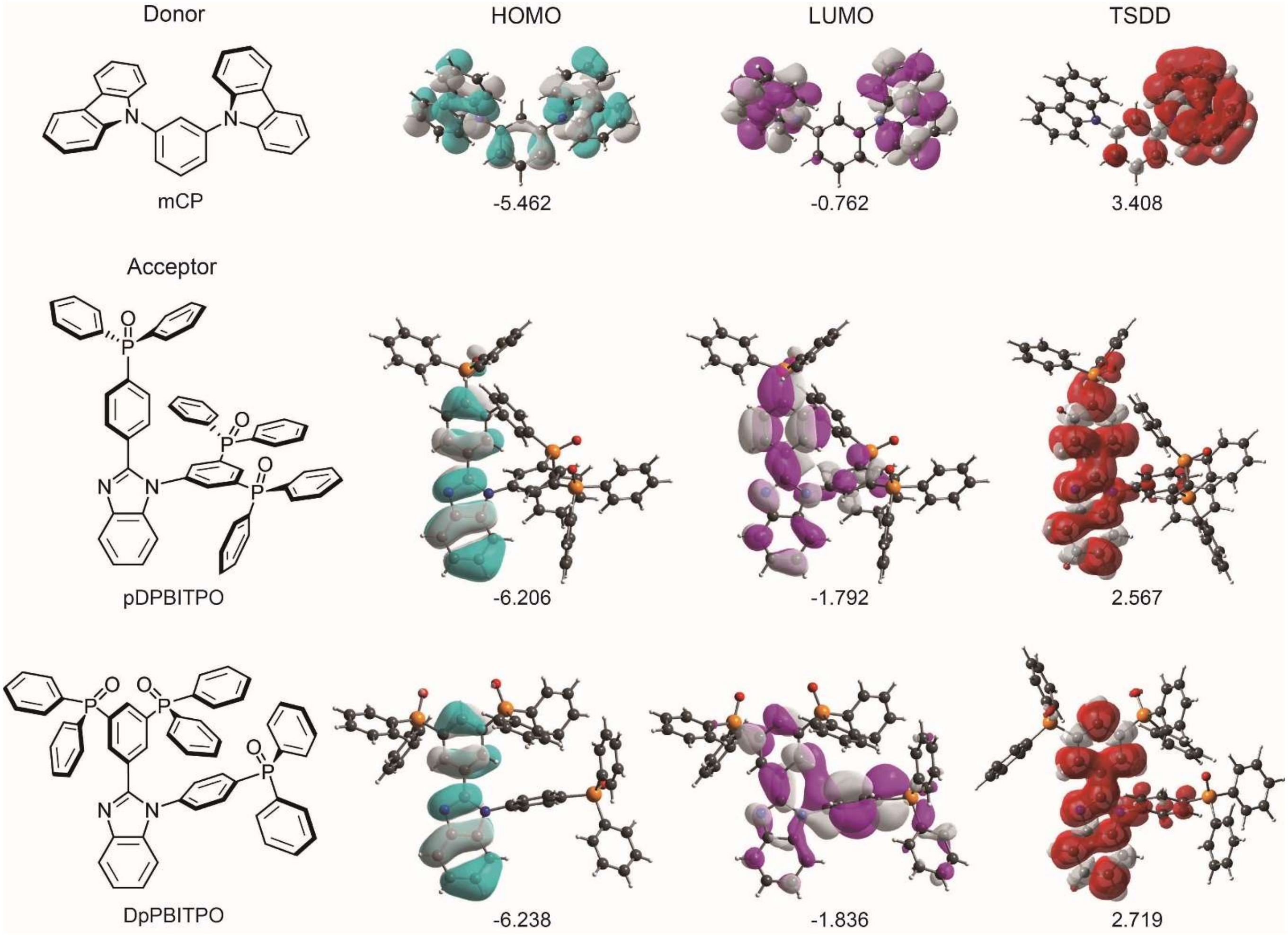

Supplementary Fig. 2 DFT simulations of molecular structures of mCP, pDPBITPO and DpPBITPO and associated contours and energy levels of frontier molecular orbitals and spin-density distributions of T_1 states (TSDD). All molecules show overlapped HOMOs and LUMOs, indicating their unipolar characteristics. The relatively centralized LUMOs of PO acceptors on the phenyls linked with DPPO moieties reflect the electron-withdrawing effect of the P=O group on electron injection to the molecules. The HOMO energy gaps between mCP and PO acceptors are ~0.8 eV, comparable to their LUMO gaps of ~1.0 eV. The HOMO-LUMO gap of mCP is ~4.7 eV, similar to ~4.5 eV of PO acceptors. Comparable charge trap depths and HOMO-LUMO gaps of these donor and acceptor molecules can facilitate electronic coupling at singlet states. The triplet configuration of mCP with a high triplet energy level of 3.4 eV is similar to its ground state. In contrast, the phenylbenzimidazole groups in pDPBITPO and DpPBITPO are coplanar at their triplet states. Such structural relaxation induces remarkably lower triplet energy levels. The large triplet energy gaps prevent triplet electronic coupling between mCP and PO acceptors.

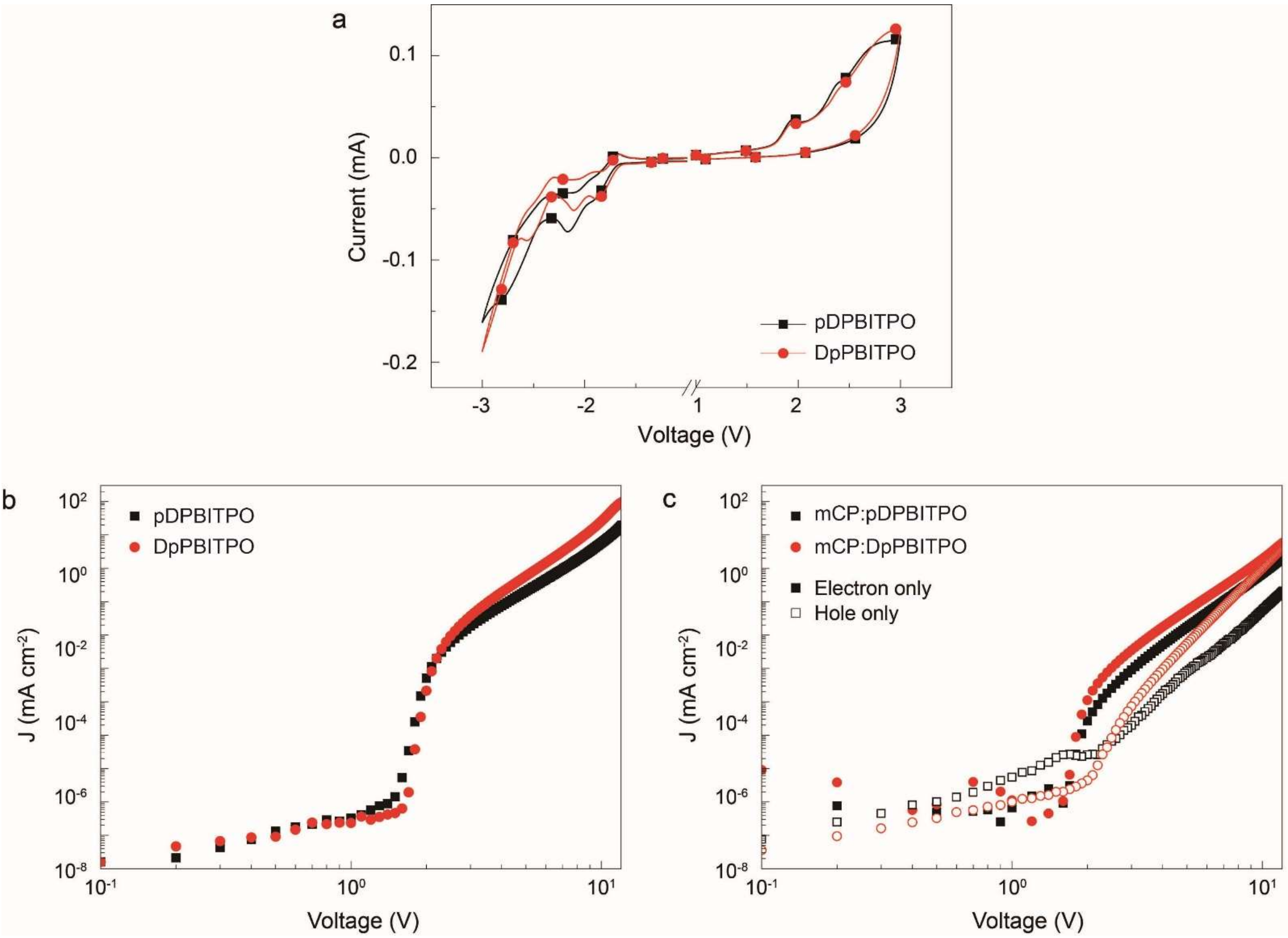

Supplementary Fig. 3 Electrical properties of pDPBITPO, DpPBITPO, mCP:pDPBITPO and mCP:DpPBITPO. **a,** Cyclic voltammograms (CV) of pDPBITPO and DpPBITPO, measured at room temperature in a nitrogen atmosphere with 100 mV s^{-1} scanning rate. **b,** IV characteristics of pDPBITPO and DpPBITPO-based, electron-only devices with a configuration of ITO|LiF (1 nm)|PO (100 nm)|LiF (1 nm)|Al (100 nm). **c,** IV characteristics of mCP:pDPBITPO and mCP:DpPBITPO-based, single-carrier transporting devices with configurations of ITO|MoO_3 (6 nm)|Exciplex (100 nm)|MoO_3 (6 nm)|Al (100 nm) for hole-only and ITO|LiF (1 nm)|Exciplex (100 nm)|LiF (1 nm)|Al (100 nm) for electron-only, respectively. According to onset voltages of the redox peaks in CV curves, the HOMO and LUMO energy levels of pDPBITPO and DpPBITPO are -6.5 and -3.1 eV, respectively, for effective electron injection and hole blocking (Table S1). By fitting IV curves of the nominal electron-only devices with a space charge-limited current (SCLC) model, electron mobilities (μ_e) of pDPBITPO and DpPBITPO are estimated at the level of 10^{-4} cm^2 V^{-1} s^{-1}, making them competent as electron-transporting materials (Table S1). In contrast to their PO acceptors, μ_e of mCP:pDPBITPO and mCP:DpPBITPO is roughly halved, helping confine electrons in emissive layers for recombination (Table S2). Meanwhile, mCP:DpPBITPO reveals a more balanced carrier transporting capacity with a hole mobility (μ_h) of ~3 x 10^{-5} cm^2 V^{-1} s^{-1}, which is one order of magnitude larger than that of mCP:pDPBITPO.

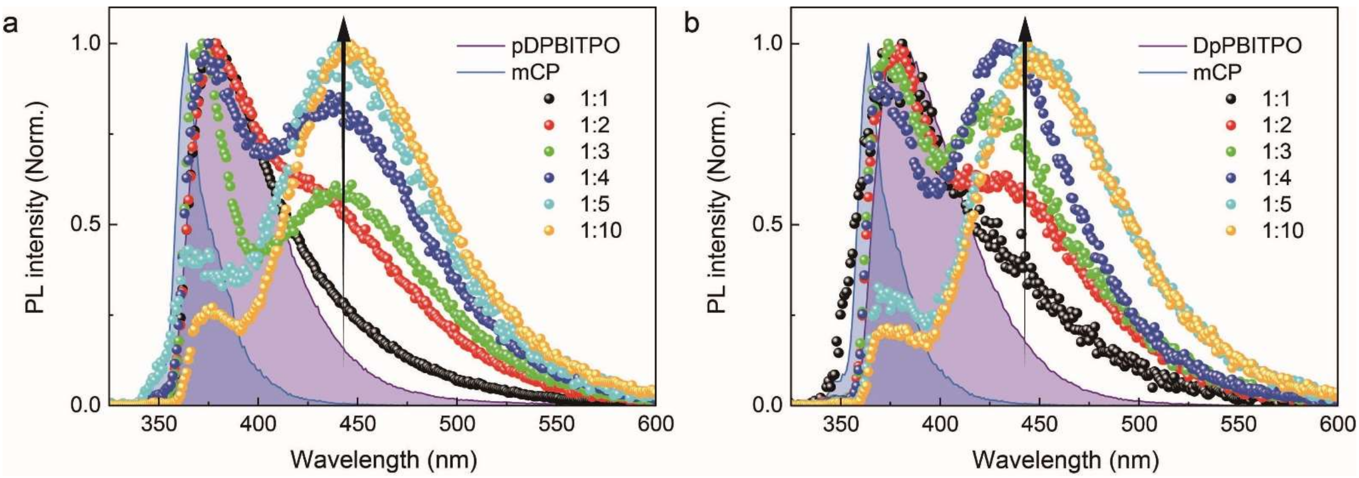

Supplementary Fig. 4 PL spectra of dichloromethane solutions of mCP and PO acceptors (**a,** pDPBITPO and **b,** DpPBITPO) with different ratios. The solution concentration of PO acceptors is 0.1 mol L^{-1}. The mCP solution (0.1 mol L^{-1}) is gradually added to the PO acceptor solutions. Increasing mCP concentration generates a new emission peak around 450 nm and decreases the emission intensity of PO acceptors. Compared to the emission of mCP and PO acceptors, new emission bands correspond to exciplex emission, with a large red-shift of ~70 nm. Therefore, charge transfer between the mCP donor and PO acceptors induces the formation of mCP:DpPBITPO and mCP:pDPBITPO exciplexes. Furthermore, For all ratios, the emission from acceptors can be observed, reflecting limited donor-acceptor interactions.

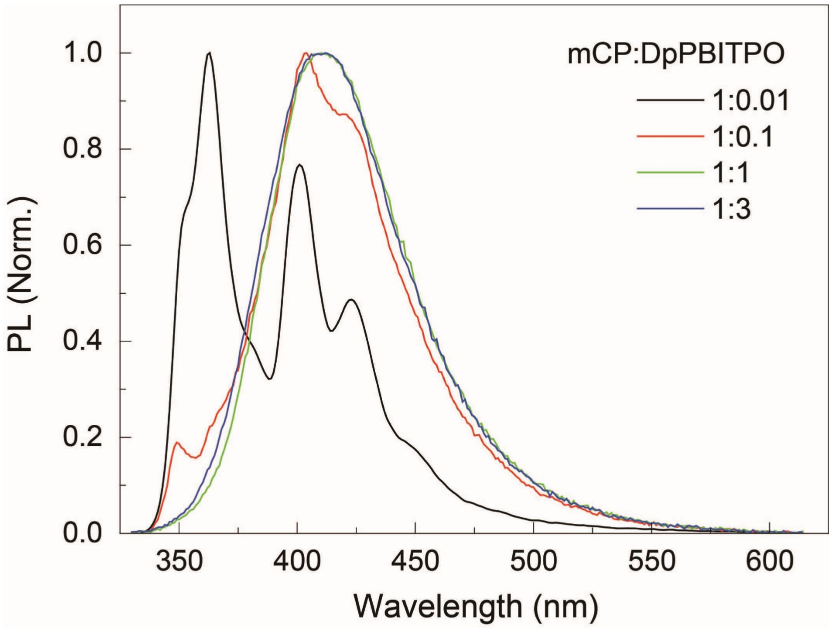

Supplementary Fig. 5 PL spectra of vacuum-evaporated mCP:DpPBITPO films with different donor/acceptor molar ratios. Even at a low DpPBITPO ratio of 0.01, new emission peaks are generated at wavelengths beyond 400 nm. Upon increasing the DpPBITPO ratio to 0.1, deep-blue peaks around 400 nm become predominant, accompanied by weakened emissions of mCP at 350 nm. The fine structure of newly generated peaks is due to diverse donor-acceptor interactions at low DpPBITPO concentrations. Further increasing the mCP/DpPBITPO ratio to 1:1 gives rise to smooth, broad, structureless emissions centered at 413 nm, accompanied by the disappearance of mCP peaks. The emission bathochromic shift and broadening upon increasing the concentration of DpPBITPO indicate the gradual formation of the mCP:DpPBITPO exciplex.

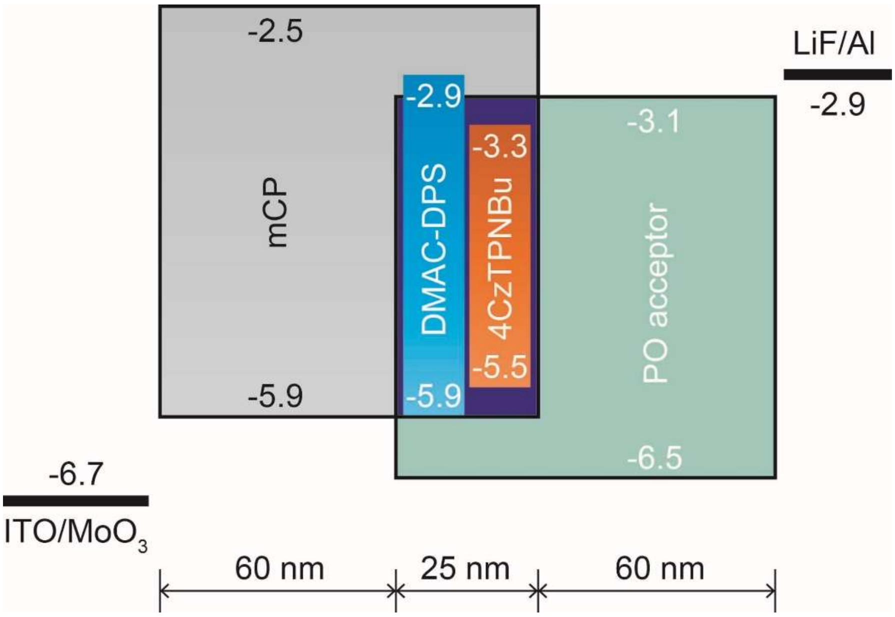

Supplementary Fig. 6 Configuration and energy-level diagram of the trilayer devices comprising ITO|MoO_3 (6 nm)|mCP (60 nm)|EML (25 nm)|Acceptor (60 nm)|LiF (1 nm)|Al (100 nm). Comparable HOMO and deeper LUMO energy levels of exciplex hosts suggest an energy transfer-based electroluminescent mechanism dominated by DMAC-DPS.

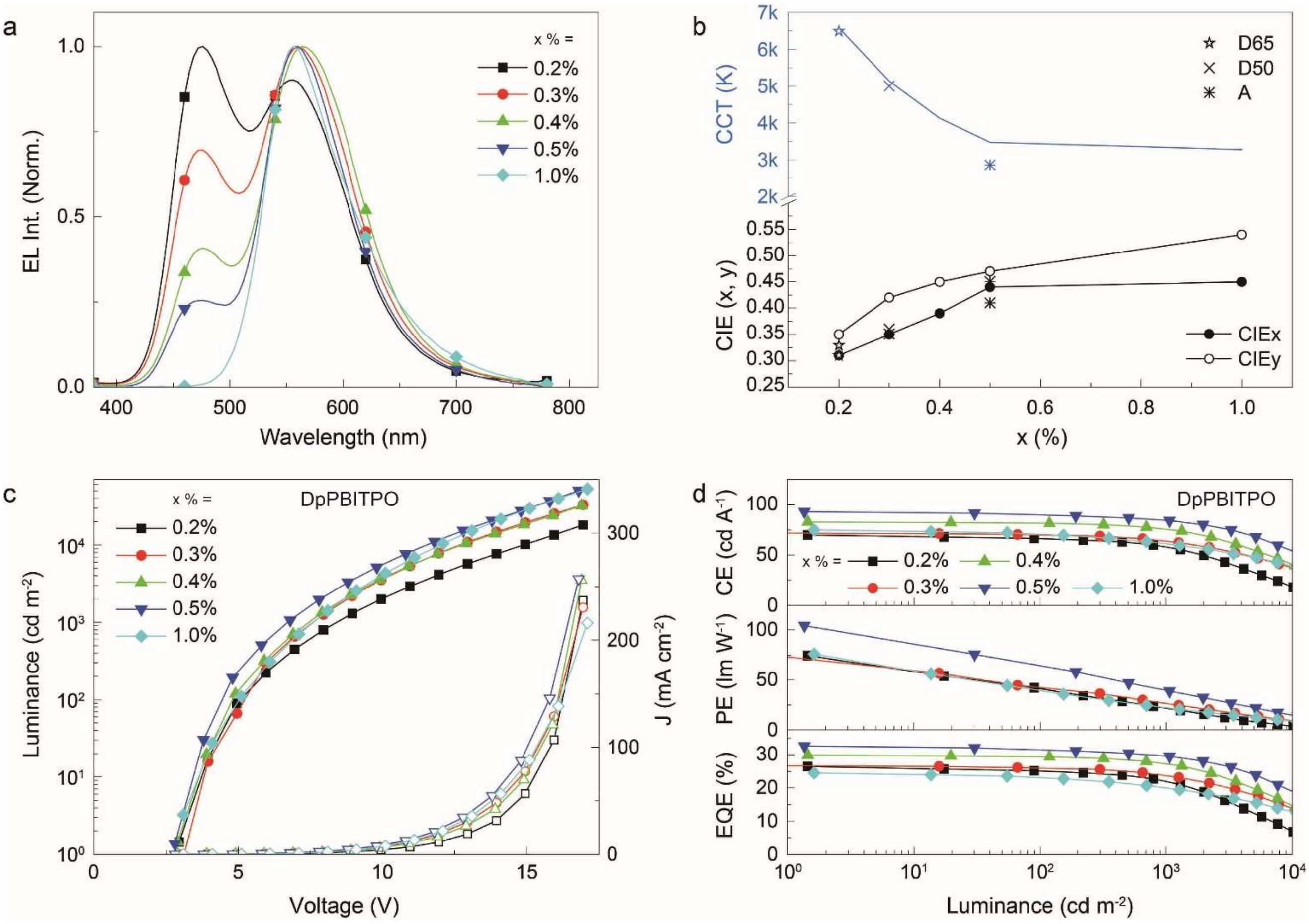

Supplementary Fig. 7 Electroluminescence characterizations of TADF WOLEDs with emissive layers of mCP:DpPBITPO:35% DMAC-DPS:x% 4CzTPNBu (x = 0.2, 0.3, 0.4, 0.5 and 1.0). **a,** Doping concentration-dependent EL variation of as-fabricated devices. **b,** Variation tendency of CIE coordinates and correlated color temperature (CCT) with increasing doping concentrations. Values of standard illuminants D65, D50, and A are marked for comparison. **c,** Voltage-*J*-luminance curves of the devices. **d,** Luminance *vs*. efficiency correlation of the devices. Upon increasing the doping concentration, the blue component intensity gradually decreased and disappeared at x = 1.0, due to enhanced energy transfer from DMAC-DPS to 4CzTPNBu. The gradually changed EL spectra also reflect that exciton allocation in the emissive layers depends mainly on energy transfer rather than direct charge/exciton capture. It shows that with increasing concentration from 0.2% to 0.5%, the CIE coordinates of the devices moved along the Planckian locus in the chromaticity diagram, corresponding to emission color change from pure white to warm white (Fig. 3b). CIE coordinates and CCT values of these WOLEDs with x = 0.2, 0.3 and 0.5 are (0.31, 0.35) and 6582 K, (0.35, 0.42) and 5136 K, and (0.44, 0.47) and 3474 K, which are close to the Illuminants D65 ((0.31, 0.33)/6499 K), D50 ((0.35, 0.36)/5003 K) and A ((0.45, 0.41)/2856 K), corresponding to average daylight, midday sunlight, and incandescent light, respectively. Significantly, all these WOLEDs display state-of-the-art performance, including turn-on voltages less than 3.0 V, maximum η_{PE} and η_{EQE} values beyond 70 lm W^{-1} and 25%, and reduced roll-offs (Supplementary Table 3). Based on the same single-EML structure, mCP:DpPBITPO enables pure white and warm white OLEDs with high-quality white light, large luminance, high $\eta_{PE,}$ and the η_{IQE} of ~100%, which are prerequisites for daily lighting applications.

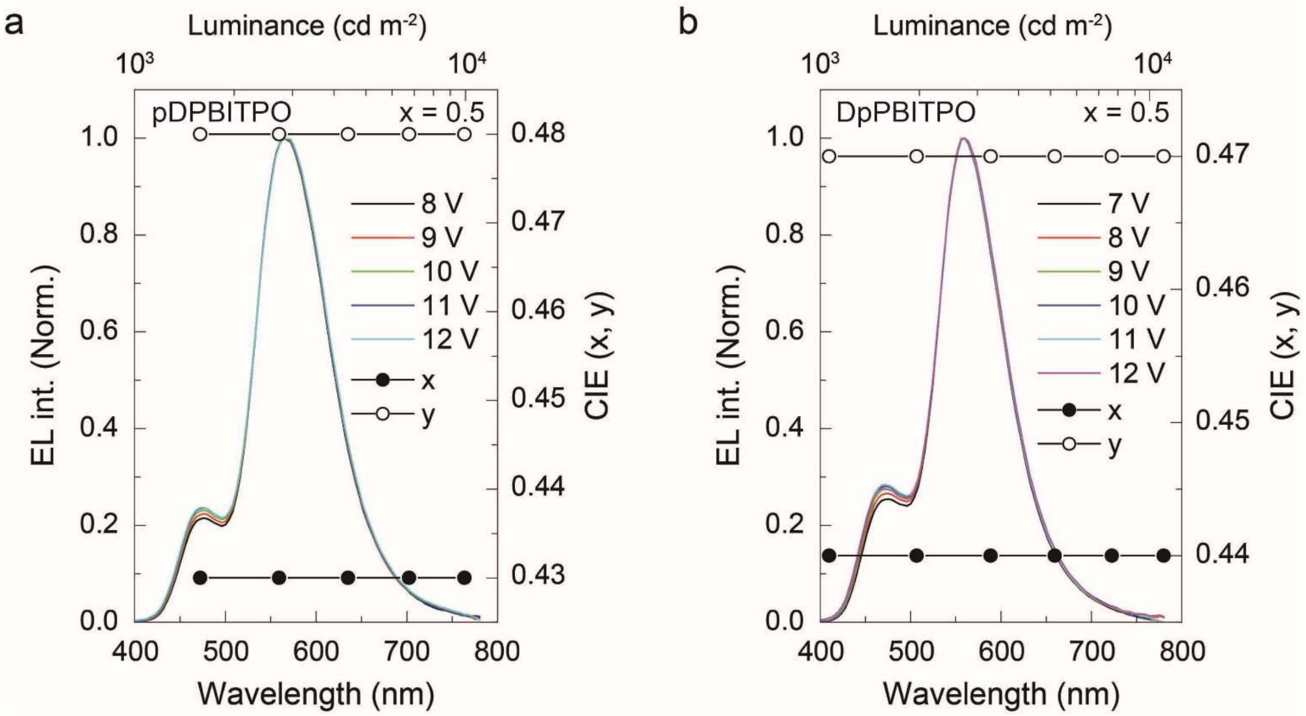

Supplementary Fig. 8 Chromatic stability of WOLEDs with emissive layers of mCP:PO acceptor: 35% DMAC-DPS:0.5% 4CzTPNBu. EL spectra and CIE coordinates of WOLEDs based on (**a**) mCP:pDPBITPO and (**b**) mCP:DpPBITPO in the luminance range of 1000 to 10000 nits. With increasing voltage, the ratio variations of blue and yellow components are almost negligible, rendering unchanged CIE coordinates. This can be attributed to stable and balanced exciton allocation for blue and yellow emissions at high exciton concentrations. Therefore, optimal, stepwise energy transfer in the emissive layers is responsible for the chromatic stability of these devices in indoor and outdoor lighting applications.

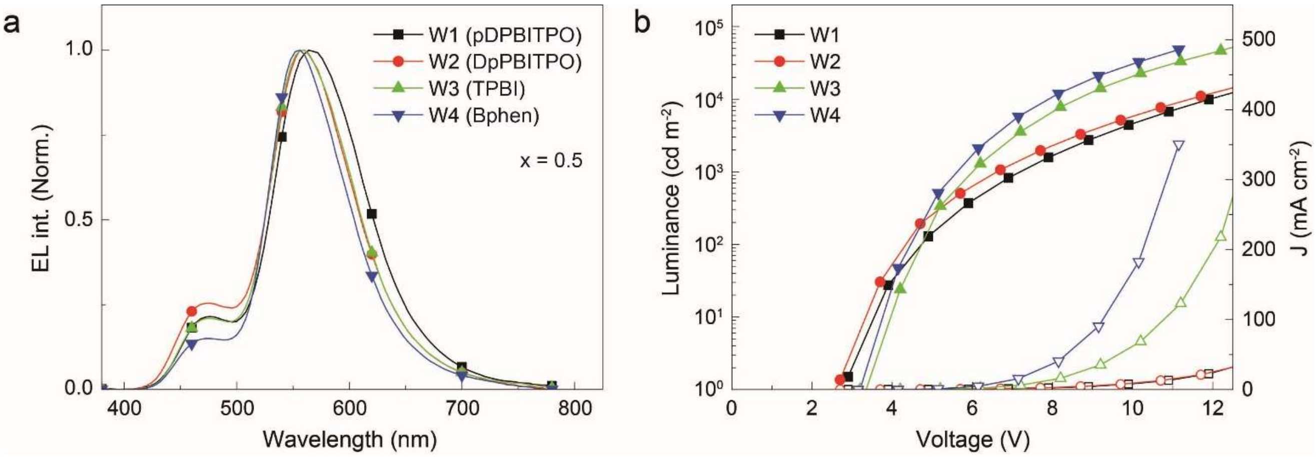

Supplementary Fig. 9 Electroluminescence characterizations of WOLEDs with emissive layers of mCP:acceptor:35% DMAC-DPS:0.5% 4CzTPNBu. **a,** EL spectra of the devices. **b,** Voltage-*J*-luminance curves of the devices. The EL spectra of mCP:TPBI-based W3 overlapped with mCP: pDPBITPO-based W1, but the blue component in the EL spectra of mCP:BPhen-based W4 was weaker, indicating more exciton allocation to 4CzTPNBu. The electron mobility of Bphen and TPBI is higher than that of pDPBITPO and DpPBITPO. Therefore, the current densities of these WOLEDs are directly proportional to the mobility of the acceptors. Nevertheless, turn-on voltages of W1 and W2 were much lower than those of W3 and W4, due to suppressed exciton quenching in W1 and W2. Since the triplet states of pDPBITPO, DpPBITPO, TPBI, and Bphen can serve as intermediate energy levels for triplet energy transfer to 4CzTPNBu, the triplet states of PO acceptors, embedded by the peripheral DPPO groups, are protected from collisional quenching. This superiority of the PO acceptors is crucial for alleviating triplet quenching during the stepwise energy transfer process, giving rise to the dramatically improved device efficiencies of W1 and W2 (Fig. 3d).

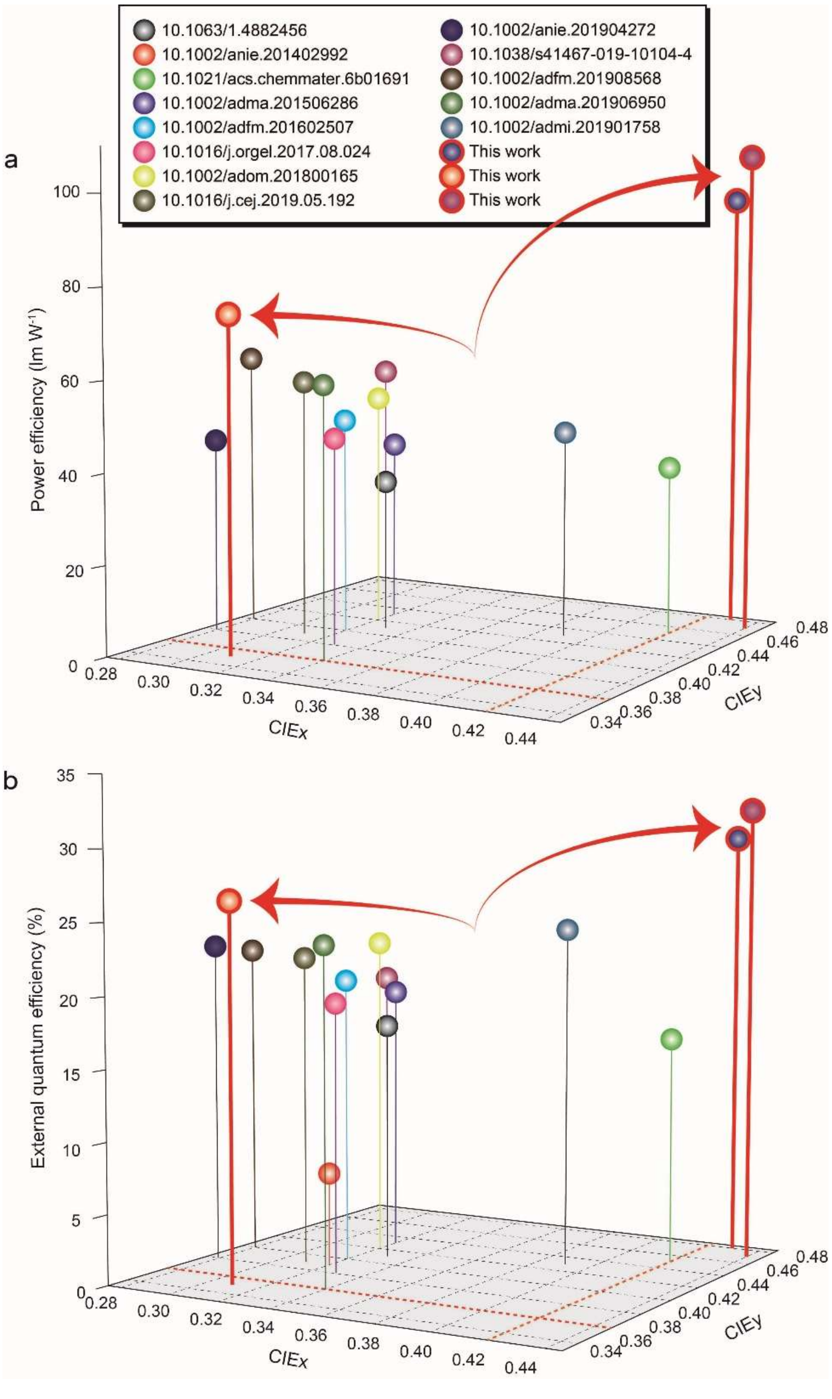

Supplementary Fig. 10 Efficiency milestones of TADF WOLEDs. **a**, η_{PE} *vs.* CIE coordinate correlation, plotted for representative TADF WOLEDs. **b**, η_{EQE} *vs.* CIE coordinate correlation, plotted for representative TADF WOLEDs. The legends show literature sources. In previous examples of TADF WOLEDs, the maximum η_{PE} and η_{EQE} are in the ranges of 40-60 lm W^{-1} and 15-25%, respectively, which are much lower than the values reported here (108.2 lm W^{-1} and 32.7%). In addition, most previously reported TADF WOLEDs have limited color purity with CIEx < 0.44 and CIEy > 0.36, deviating from pure white (0.31, 0.33) and warm white (0.45, 0.41). In contrast, the CIE coordinates of mCP:DpPBITPO-based WOLEDs can be tuned to (0.31, 0.35) and (0.44, 0.47).

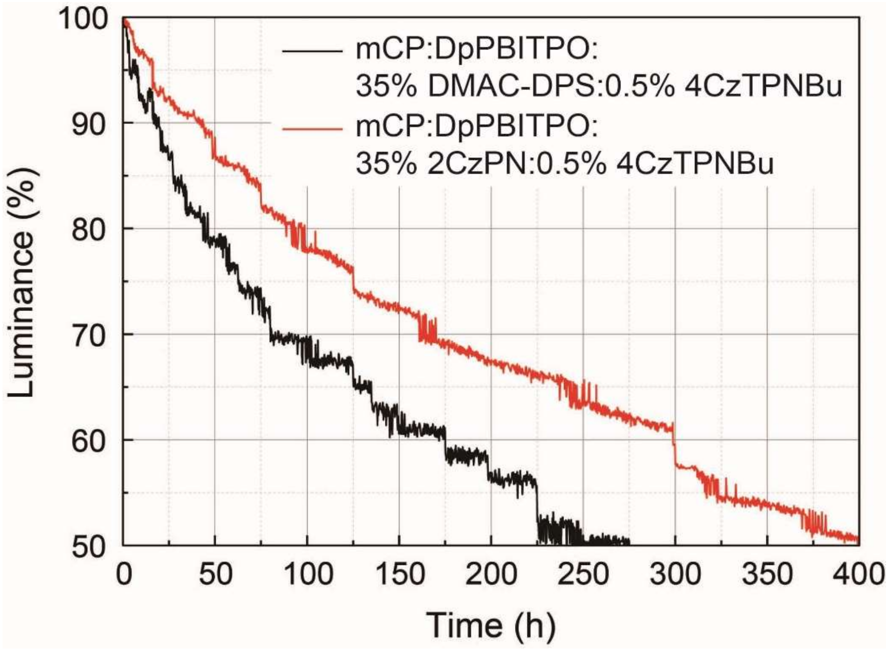

Supplementary Fig. 11 Device lifetime of WOLEDs based on mCP:DpPBITPO:35% DMAC-DPS:0.5% 4CzTPNBu and mCP:DpPBITPO:35% 2CzPN:0.5% 4CzTPNBu. Device duration was measured with an initial luminance of 1000 cd m^{-2} in a glove box in constant-current mode. Adopted devices were 0.3 cm × 0.3 cm and encapsulated with epoxy glue. Durations of the devices at 90, 75 and 50% of the initial luminance were 20, 64 and 233 hrs for mCP:DpPBITPO:35%DMAC-DPS:0.5 %4CzTPNBu based device, and 48, 125 and 422 hrs for mCP:DpPBITPO:35%2CzPN:0.5 %4CzTPNBu based device, respectively, corresponding to lifetimes of T_{90}, T_{75} and T_{50}. These values are comparable to the best results reported for full-TADF WOLEDs[17]. Remarkably long lifetimes of mCP:DpPBITPO:35%2CzPN:0.5 %4CzTPNBu based device is due to higher stability of 2CzPN than DMAC-DPS.

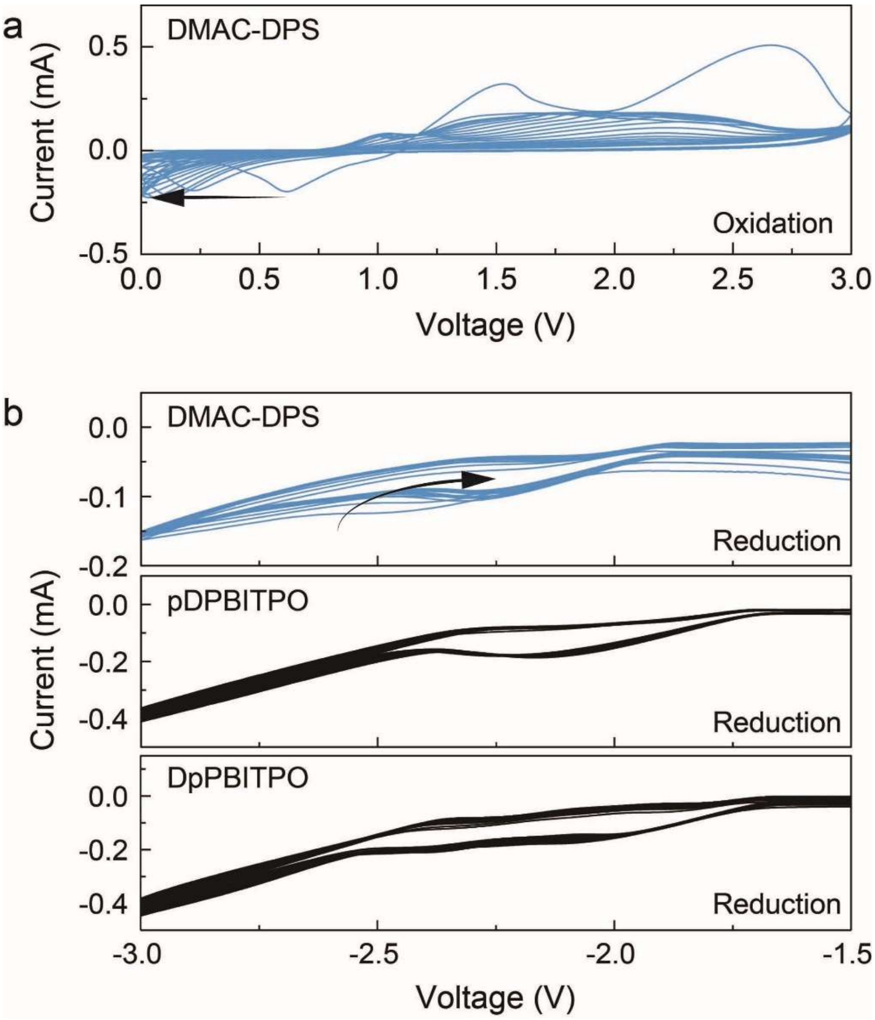

Supplementary Fig. 12 Electro-stability of DMAC-DPS and PO acceptors. **a,** Measured oxidation cyclic voltammogram of DMAC-DPS in dichloromethane (10 mg mL^{-1}). **b,** Reduction cyclic voltammograms of DMAC-DPS, pDPBITPO and DpPBITPO measured in tetrahydrofuran (10 mg mL^{-1}). Curves were measured in a nitrogen atmosphere with a scan rate of 100 mV s^{-1}. Redox peaks of DMAC-DPS shifted in large ranges during 20 cycles. DMAC-DPS is fragile during the electrochemical reaction, which induces decomposition and generates oligomer from fragment radicals. In contrast, reduction curves of PO acceptors remain almost unchanged during 20 cycles, reflecting the high electro-stability of PO acceptors during the electron gain-loss process.

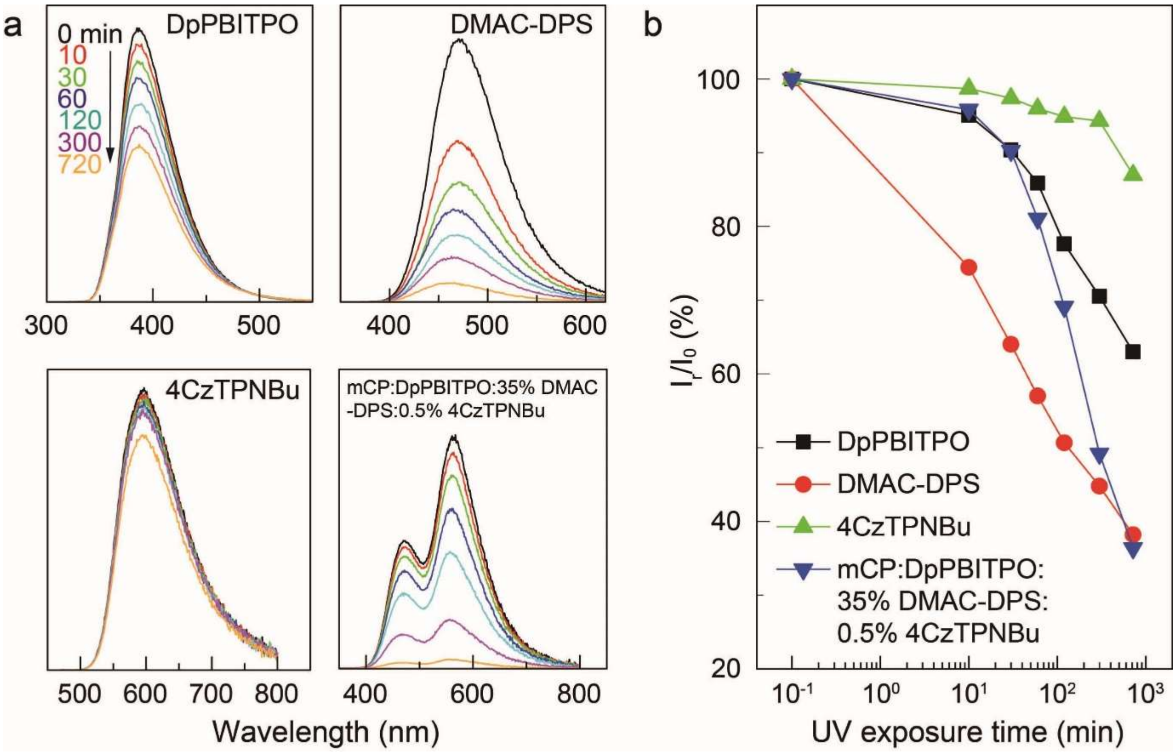

Supplementary Fig. 13 Photostability of DpPBITPO, DMAC-DPS and 4CzTPNBu neat films and mCP:DpPBITPO:35%DMAC-DPS:0.5 %4CzTPNBu film. (**a**) PL spectra of films after exposure under 365-nm UV light for 0-720 min; (**b**) correlations between the I_r/I_0 ratio of films and the exposure time. I_r/I_0 ratio is calculated according to:

$$\frac{I_r}{I_0} = \frac{I_0 - \frac{(I - I_0) \cdot A_{PO}}{A}}{I_0}$$

where I_r is the relative PL intensity, I is the measured PL intensity, I_0 is the initial PL intensity, A_{PO} is the 365-nm absorbance of phosphine oxide acceptor, and A is the 365-nm absorbance of the material. UV exposure induces a sharp decrease in the PL intensity of DMAC-DPS film. After 720 min exposure, PL of DMAC-DPS film nearly completely vanished, with a I_r/I_0 ratio of as small as 38%. In contrast, after 720-min exposure, PL intensity of DpPBITPO film keeps 63% of I_0, and the I_r/I_0 ratio of 4CzTPNBu (87%) is the biggest. Furthermore, for mCP:DpPBITPO:35%DMAC-DPS:0.5%4CzTPNBu film, before 30-min exposure, its I_r/I_0 ratio is similar to that of neat DpPBITPO film. However, from 30 to 720 min, its I_r/I_0 variation becomes similar to that of DMAC-DPS. It means before 30-min exposure, most of the UV light was absorbed by the matrix. After 30 min, the matrix absorption saturates, and DMAC-DPS becomes involved in absorption, inducing sharply decreased I_r/I_0. Photodegradation of mCP:DpPBITPO:35%DMAC-DPS:0.5%4CzTPNBu film is mainly attributed to the photochemically unstability of DMAC-DPS.

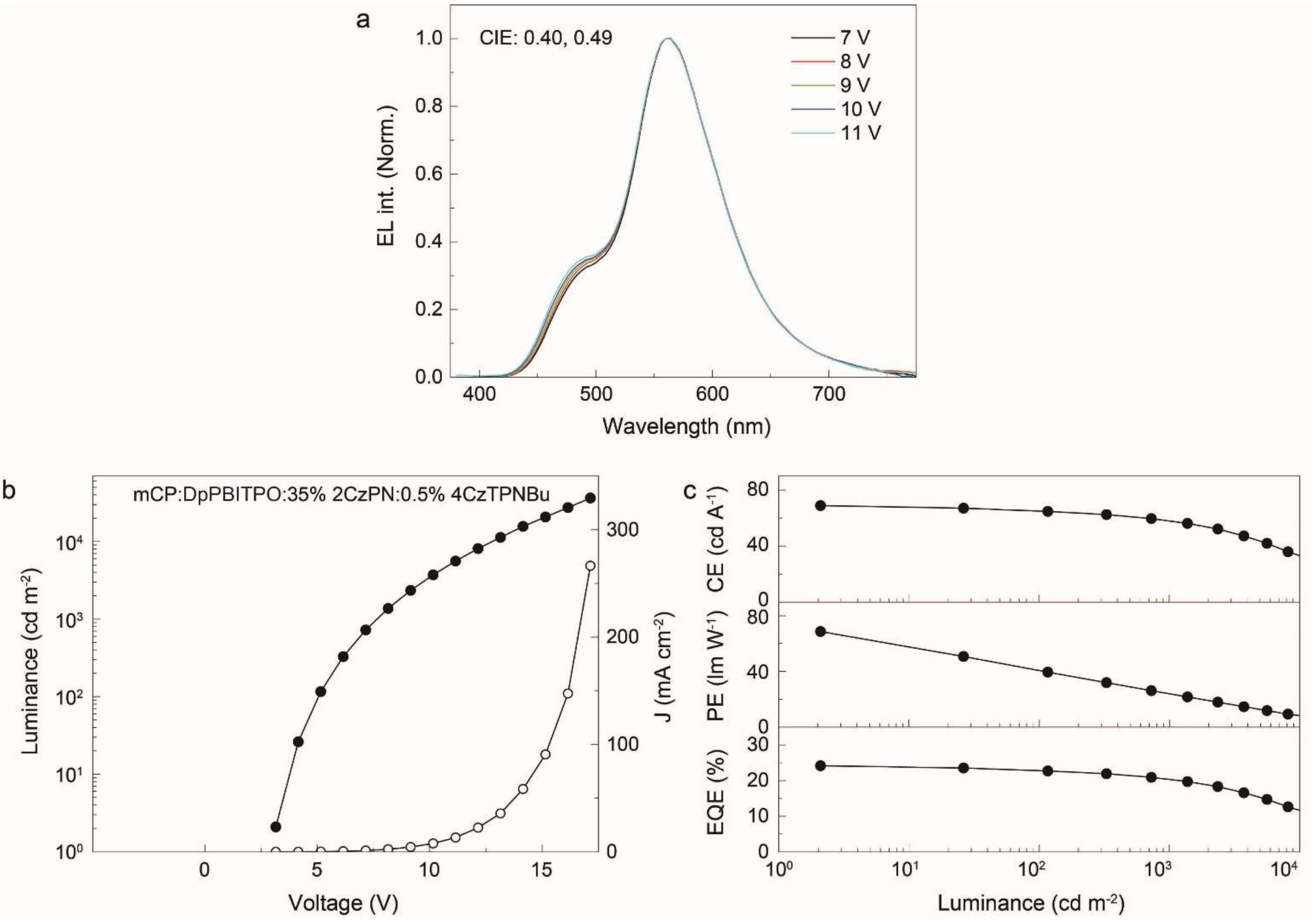

Supplementary Fig. 14 Electroluminescence characterizations of WOLEDs with mCP:PO acceptor:35%2CzPN:0.5%4CzTPNBu. **(a)** EL spectra at 7-11 V. **(b)** Voltage-*J*-luminance curves. **(b)** Luminance *vs*. efficiency correlation of the devices. EL spectrum includes two peaks from 2CzPN and 4CzTPNBu, respectively. The turn-on voltage of this device is 3.15 V at 1 cd m^{-2}. The maximum efficiencies reached 68.9 cd A^{-1}, 68.7 lm W^{-1} and 24.2%, respectively.

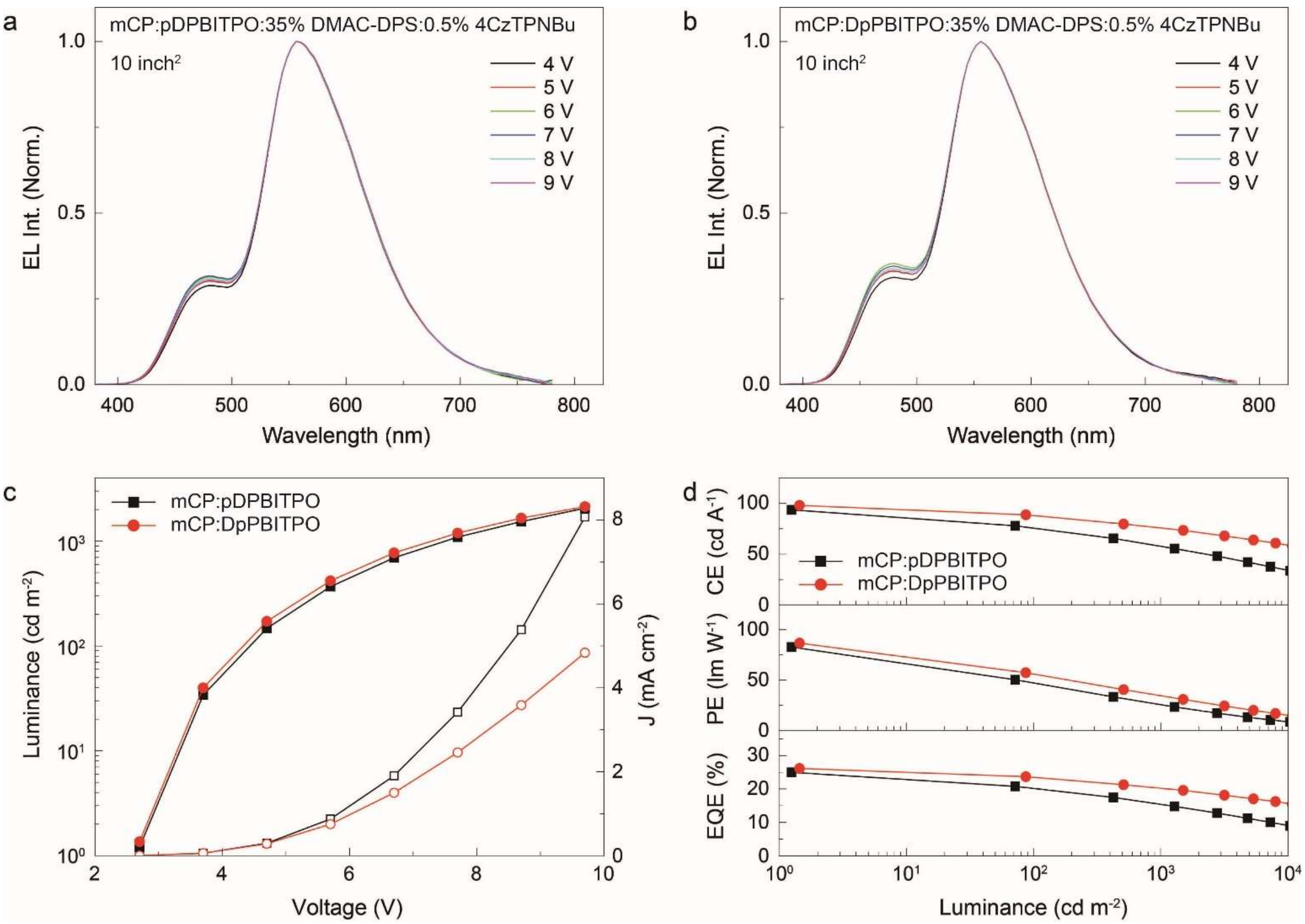

Supplementary Fig. 15 Electroluminescence characterizations of 10-inch^2 TADF WOLEDs with mCP:PO acceptor:35%DMAC-DPS:0.5%4CzTPNBu. EL spectra of (**a**) mCP:pDPBITPO- and (**b**) mCP:DpPBITPO-based devices at 4-9 V. **c,** Voltage-*J*-luminance curves. **d,** Luminance *vs*. efficiency correlation of the devices. The 10-inch^2 devices were fabricated through vacuum evaporation and directly measured at ambient conditions without encapsulation. Turn-on voltages of the two 10-inch^2 devices were slightly lower than those of the 0.09-cm^2 basic unit. Power efficiencies of the 10-inch^2 devices were much higher than achieved by previously reported TADF WOLEDs (for benchmarking, see Supplementary Table 3).

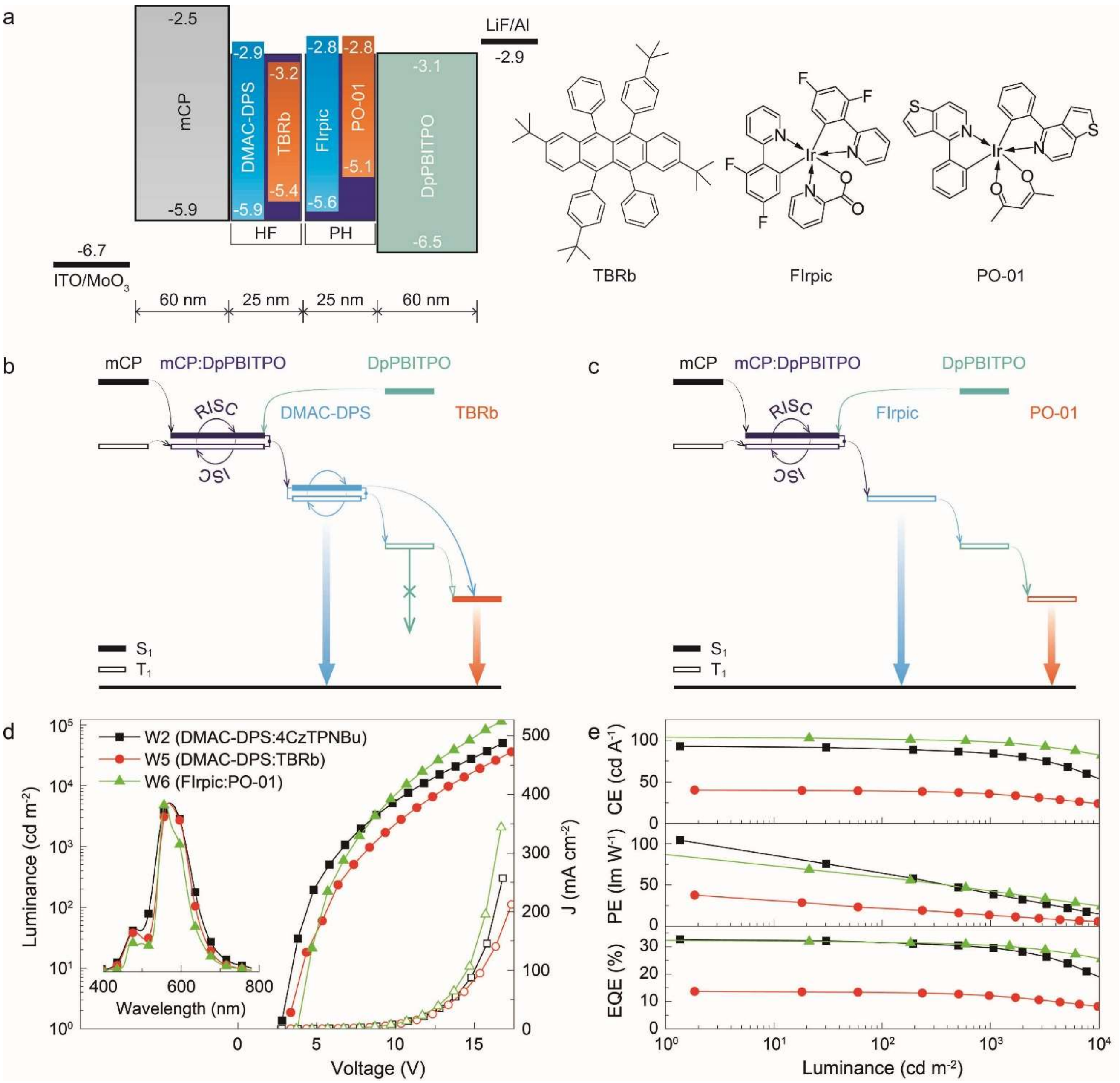

Supplementary Fig. 16 Electroluminescence characterizations of single-EML hyperfluorescent and phosphorescent WOLEDs based on mCP:DpPBITPO. **a**, Device structure and energy level diagram of WOLEDs and chemical structures of yellow fluorophore TBRb, sky-blue phosphor FIrpic and yellow phosphor PO-01. Energy level diagram and energy transfer process in (**b**) hyperfluorescent WOLED (W5) with an emissive layer of mCP:DpPBITPO:35%DMAC-DPS:0.5%TBRb, and (**c**) phosphorescent WOLED (W6) with an emissive layer of mCP:DpPBITPO:35%FIrpic:0.5%PO-01. **d,** Comparison of voltage-*J*-luminance curves of W2, W5 and W6. **d**, Luminance *vs*. efficiencies correlation of the devices. The T_1 energy level of DpPBITPO lies between the excited energy levels of DMAC-DPS and the S_1 energy level of TBRb. Since the radiative state of TBRb is S_1 state, the energy transfer to TBRb is mainly from the S_1 state of DMAC-DPS rather than from the T_1 state of DpPBITPO. Nevertheless, W5 achieved good EL performance with a maximum η_{EQE} of 13.7% and a small roll-off. On the contrary, the radiative states of the

phosphors are T_1 states; therefore, the T_1 state of DpPBITPO facilitates triplet energy transfer from FIrpic to PO-01. This case is similar to mCP:DpPBITPO-based white TADF systems. W6 achieved maximum efficiencies of 88.3 lm W^{-1} and 32.4%, comparable to the best phosphorescent WOLEDs reported so far[3]. It is noted that the blue emissive component of W6 was weaker than that of W2. For mCP:DpPBITPO:35%FIrpic:0.5%PO-01, the T_1 state of FIrpic was simultaneously involved in blue emission and blue-to-yellow energy transfer, leading to direct competition between blue phosphorescence and blue-to-yellow energy transfer. On the contrary, for mCP:DpPBITPO:35%DMAC-DPS:0.5%4CzTPNBu, blue emission is from the S_1 state of DMAC-DPS rather than the T_1 state. Incorporation of the T_1 state for DpPBITPO in triplet energy transfer optimizes triplet-exciton allocation to 4CzTPNBu, but hardly affects DMAC-DPS in singlet-exciton utilization. Thus, the exciplex-based energy relaying mechanism is universal for optimization of exciton allocation in single-layer, white-emitting systems.

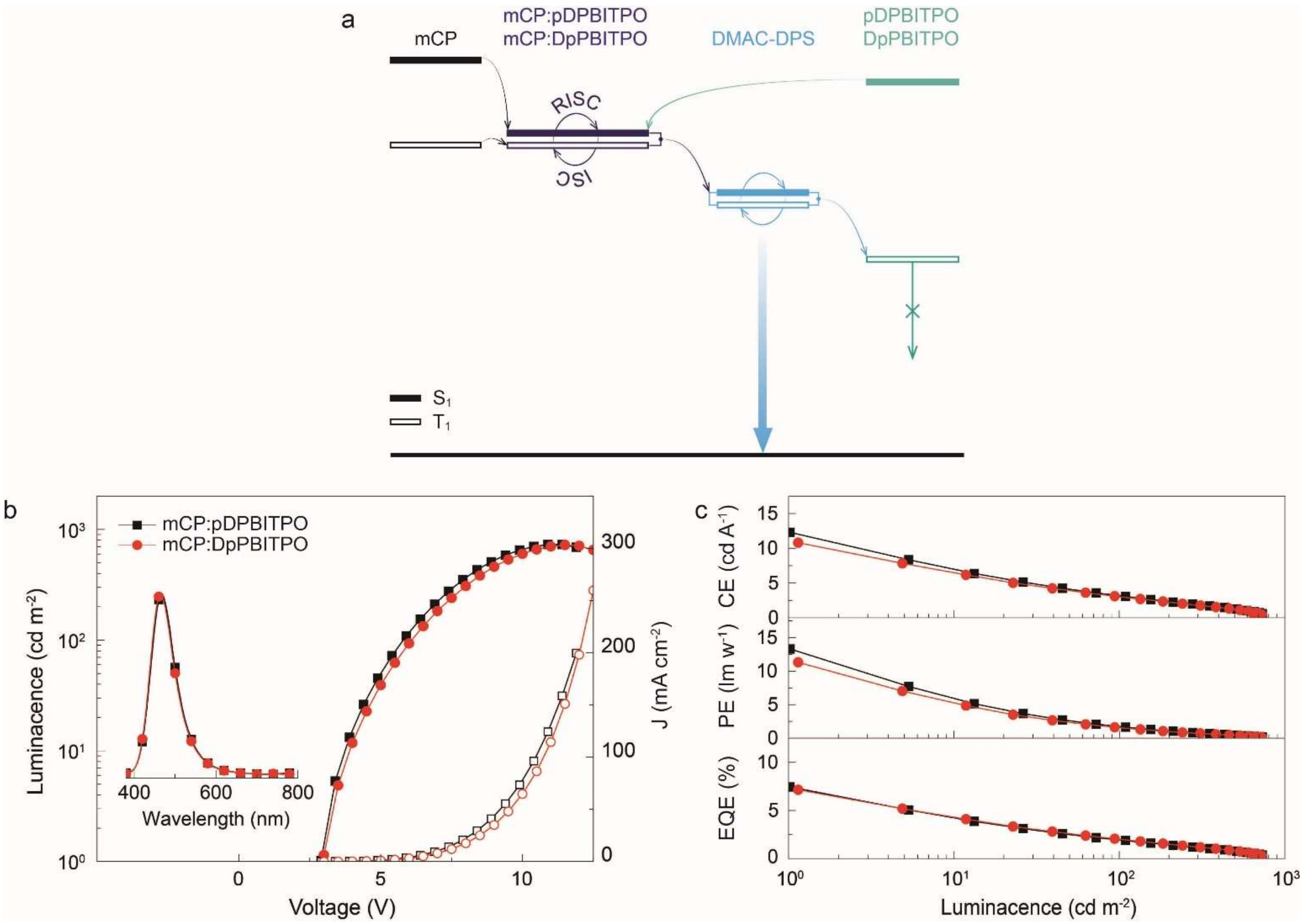

Supplementary Fig. 17 Electroluminescence characterizations of blue TADF diodes with emissive layers of mCP:PO acceptor:30%DMAC-DPS. **a,** Energy transfer process in the emissive layer. Singlet electronic coupling between mCP and PO acceptors generates S_1 energy levels for the exciplexes, while mCP and PO acceptors contribute to high-lying and low-lying triplet energy levels, respectively. **b,** Voltage-*J*-luminance curves and EL spectra (inset) of as-fabricated devices. **c,** Luminance *vs*. efficiency correlation of the devices. Excitons are first formed on exciplex molecules and then transferred to DMAC-DPS dopants. However, low-lying triplet energy levels lead to triplet exciton leakage from DMAC-DPS and nonradiative deactivation. Therefore, the blue devices displayed low EL performance, e.g., a luminance within 1000 cd m^{-2} and a maximum $\eta_{EQE} < 8\%$, far below conventional devices (> 10000 cd m^{-2} and ~20%)[20].

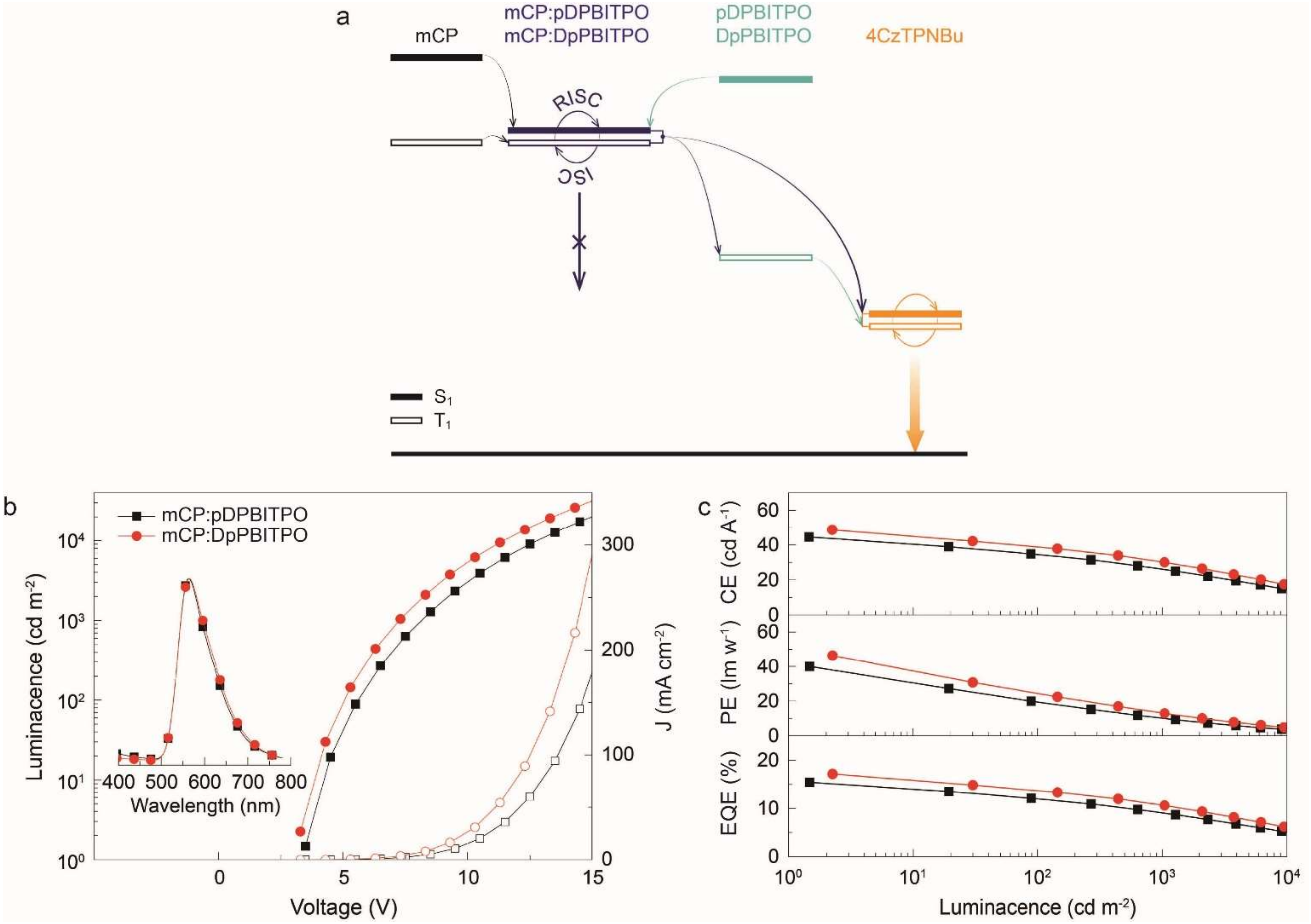

Supplementary Fig. 18 Electroluminescence characterizations of yellow TADF diodes with emissive layers of mCP:PO acceptor:5% 4CzTPNBu. **a,** Energy transfer process in the emissive layer. **b,** Voltage-*J*-luminance curves and EL spectra (inset) of the as-fabricated devices. **c,** Luminance *vs*. efficiency correlation of the devices. Since the large energy gaps between the exciplexes and 4CzTPNBu do not support efficient energy transfer, the low-lying T_1 levels of the exciplexes dominate energy transfer to 4CztPNBu. However, the population of the T_1 states is limited upon charge recombination. Nevertheless, deeper frontier molecular orbitals of 4CNTPNBu facilitate direct charge-carrier capture and recombination, alleviating inefficient host-dopant energy transfer. The maximum η_{EQE} of yellow devices was ~15%, two-thirds of the reported values for 4CzTPNBu-based devices[34]. The blue (see Supplementary Fig. 12) or the yellow device alone cannot achieve 100% exciton harvesting.

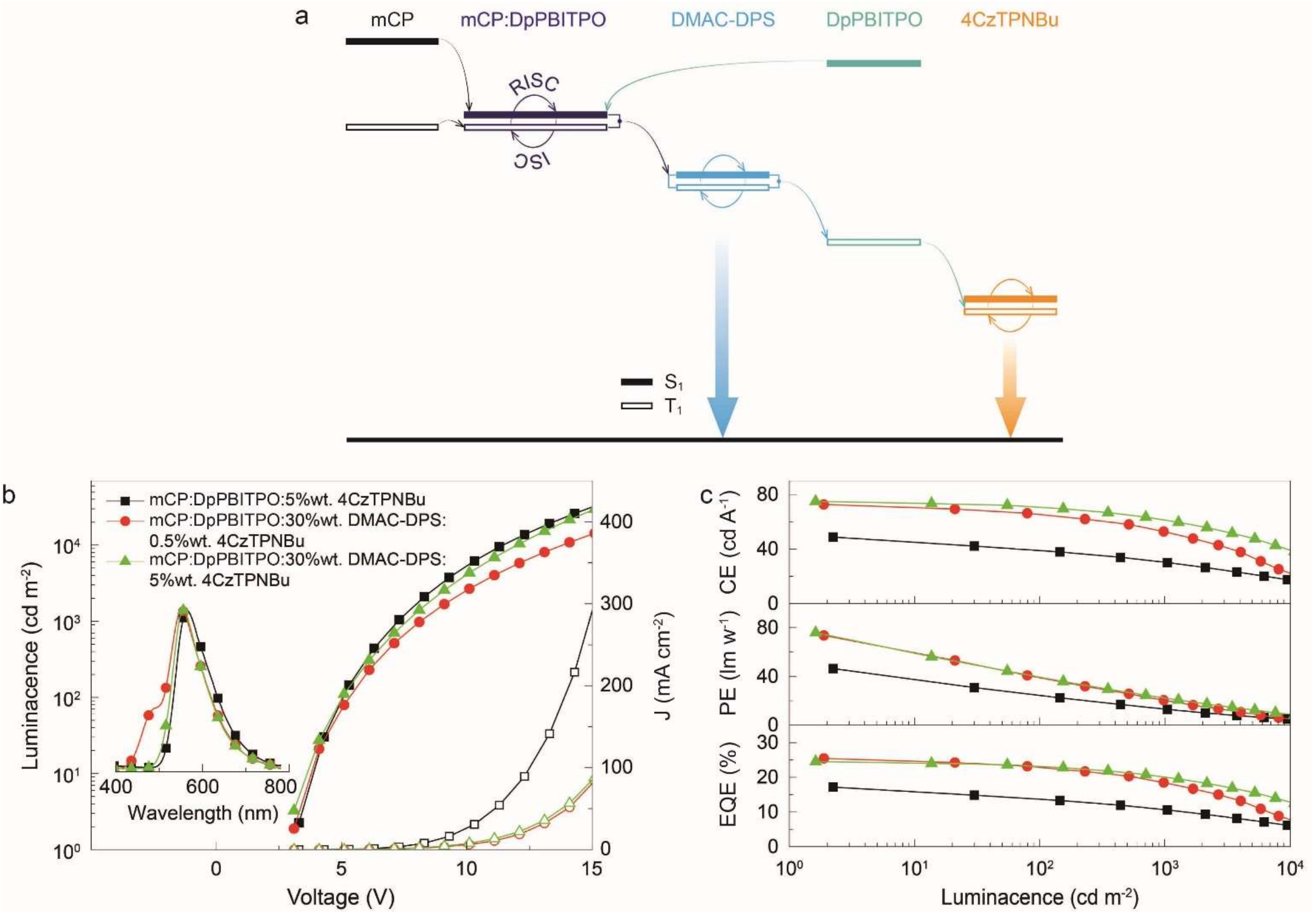

Supplementary Fig. 19 Comparison of electroluminescent properties for a singly-doped yellow TADF diode, a doubly doped white TADF diode, and a doubly doped yellow TADF diode. **a,** Energy transfer process of TADF diodes with an emissive layer of [mCP:PO acceptor]:DMAC-DPS:4CzTPNBu. **b,c,** Voltage-*J*-luminance curves and EL spectra (inset) (**b**), and luminance *vs*. efficiency correlation (**c**) of yellow and white devices with emissive layers of mCP:DpPBITPO:5%4CzTPNBu (yellow), mCP:DpPBITPO:30%DMAC-DPS:0.5%4CzTPNBu (white) and mCP:DpPBITPO:30%DMAC-DPS:5%4CzTPNBu (yellow). The 4CzTPNBu doping concentration of the yellow device with mCP:DpPBITPO:30%DMAC-DPS:5%4CzTPNBu increased 10-fold to achieve pure yellow emission. The energy transfer process of this doubly doped yellow device is the same as the white device. In contrast to the singly doped yellow devices, the incorporation of DMAC-DPS in the emissive layer improved maximum efficiencies of the doubly doped yellow devices by ~40% (75.9 lm W^{-1} and 24.5%). In doubly doped systems, triplet excitons can leak from DMAC-DPS to the low-lying T_1 level of the hosts before being utilized by 4CzTPNBu for radiation. At the same time, the T_1 level of DMAC-DPS serves an intermediate energy level between the exciplexes and 4CzTPNBu to improve energy transfer.

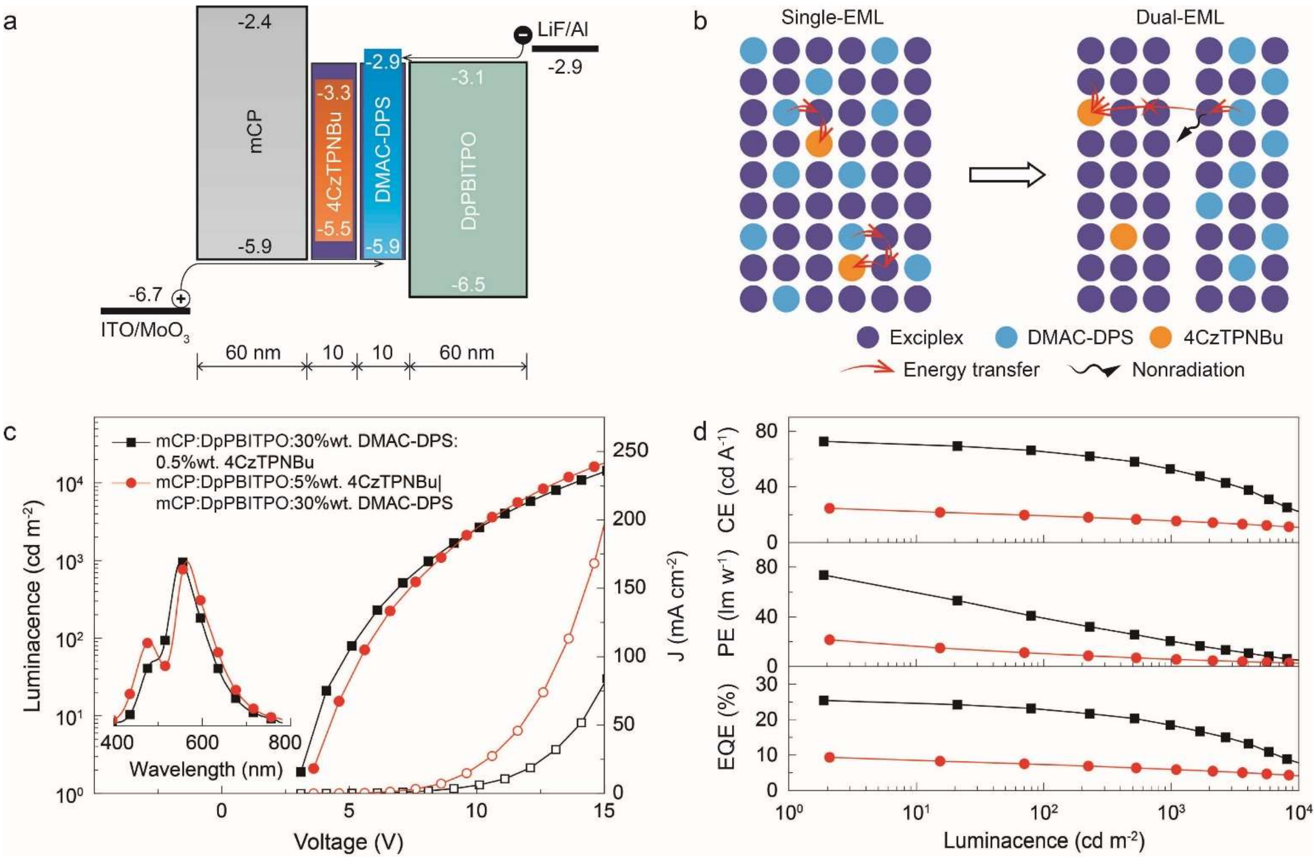

Supplementary Fig. 20 Electroluminescence characterizations of single- and dual-emissive-layer WOLEDs based on mCP:DpPBITPO. **a**, Device structure and energy level diagram of the WOLEDs with dual emissive layers of mCP:DpPBITPO:5%4CzTPNBu|mCP:DpPBITPO:30%DMAC-DPS. **b**, Comparison of the energy transfer processes of single- and dual-emissive-layer devices. **c**, Voltage-*J*-luminance curves and EL spectra (inset). **d**, Luminance *vs*. efficiency correlation. To experimentally verify the role of the T_1 levels of the hosts in the electroluminescence process, a control device containing dual emissive layers was fabricated, with DMAC-DPS and 4CzTPNBu spatially separated by mCP:DpPBITPO doping. Since the short-distance Dexter mechanism dominates the triplet energy transfer, the distance between participants in the energy transfer process of DMAC-DPS → PO acceptor → 4CzTPNBu must be short. In the dual-emissive-layer device, the short distance between DMAC-DPS-PO acceptors-4CzTPNBu cannot be simultaneously satisfied to establish a continuous energy transfer process. As a result, the blue emission of the dual-EML device is more intense than that of the single-EML devices, due to inefficient energy transfer of the former. The dual-EML device suffered from triplet leakage in the blue emissive layer and exciton nonradiative transition in the yellow emissive layer, reducing maximum efficiencies to 24.6 cd A^{-1}, 21.4 lm W^{-1} and 9.3%. These values are less than half of the maximum efficiencies achieved by single-EML devices.

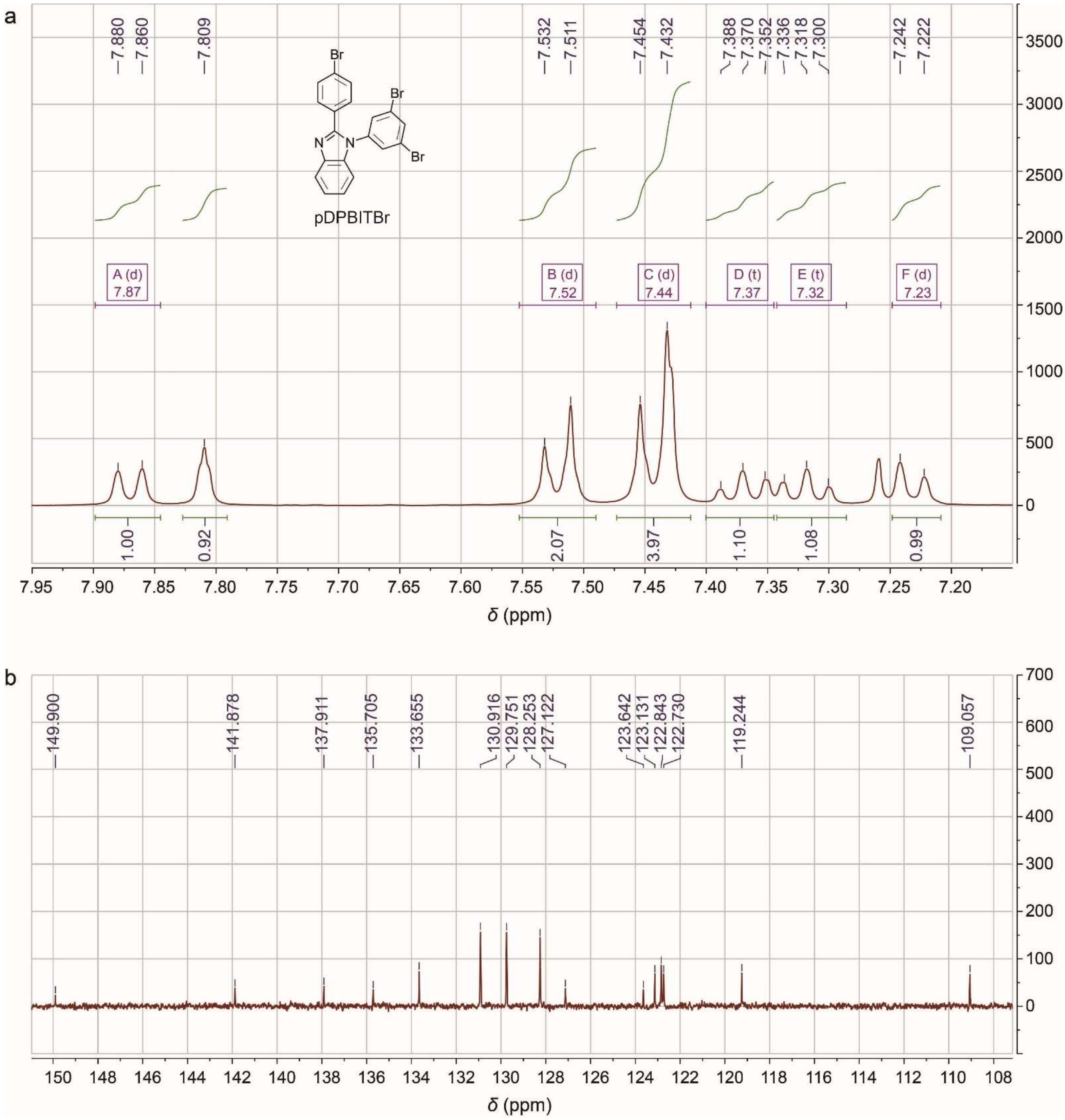

Supplementary Fig. 21 ^{1}H NMR (**a**) and ^{13}C NMR (**b**) spectra of pDPBITBr.

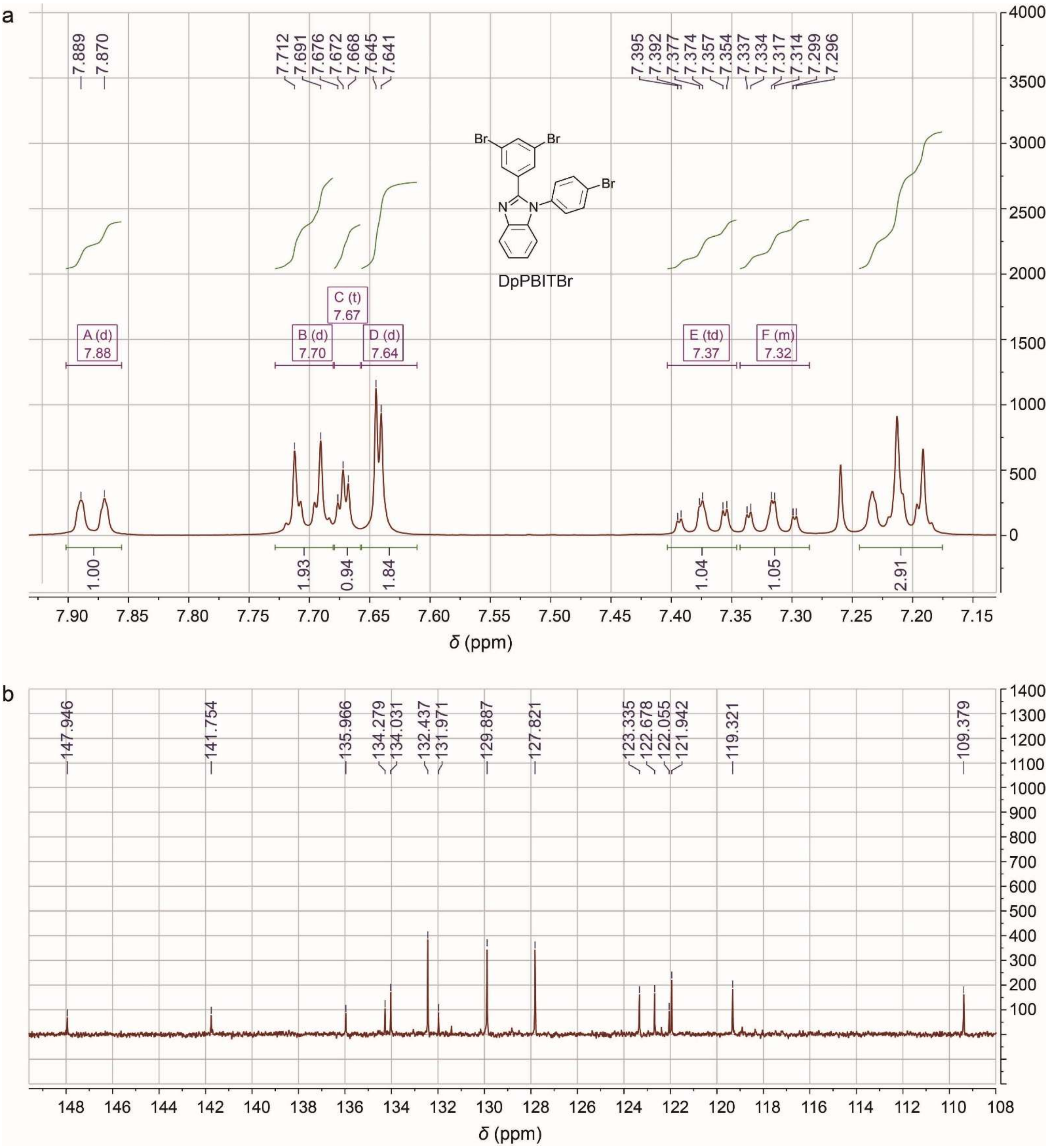

Supplementary Fig. 22 ^{1}H NMR (**a**) and ^{13}C NMR (**b**) spectra of DpPBITBr.

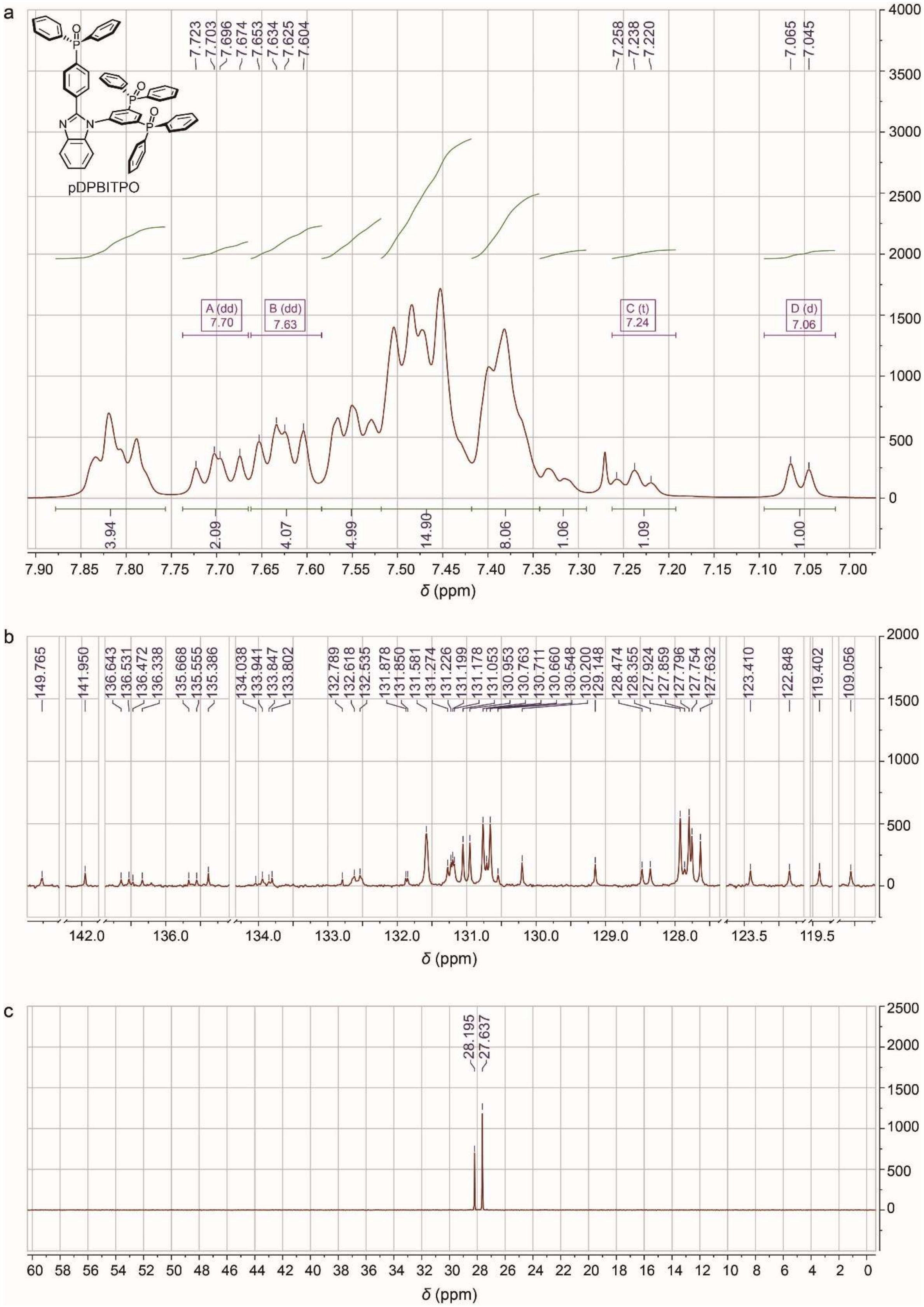

Supplementary Fig. 23 ^{1}H NMR (**a**), ^{13}C NMR (**b**) and ^{31}P NMR (**c**) spectra of pDPBITPO.

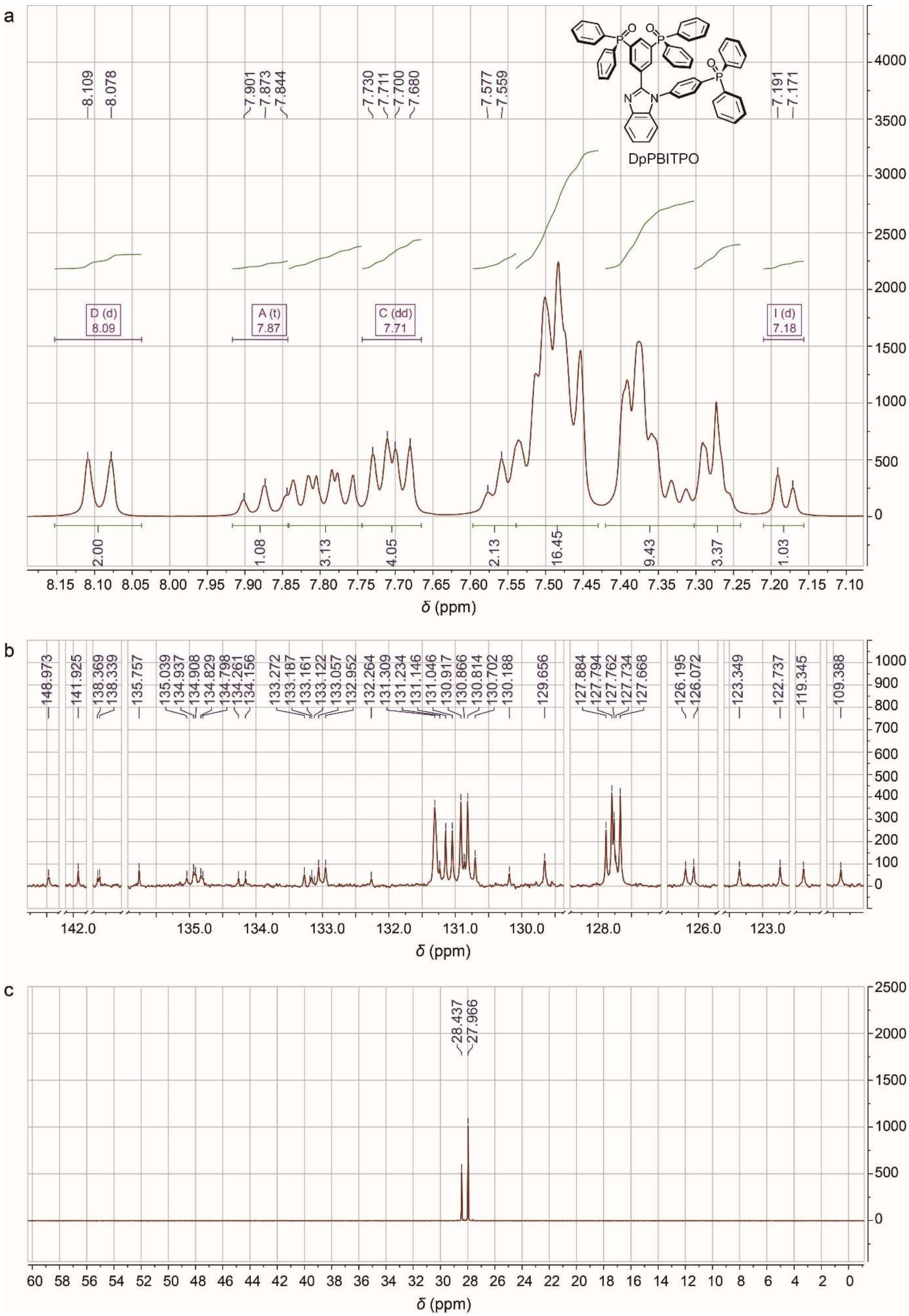

Supplementary Fig. 24 ^{1}H NMR (**a**), ^{13}C NMR (**b**) and ^{31}P NMR (**c**) spectra of DpPBITPO.

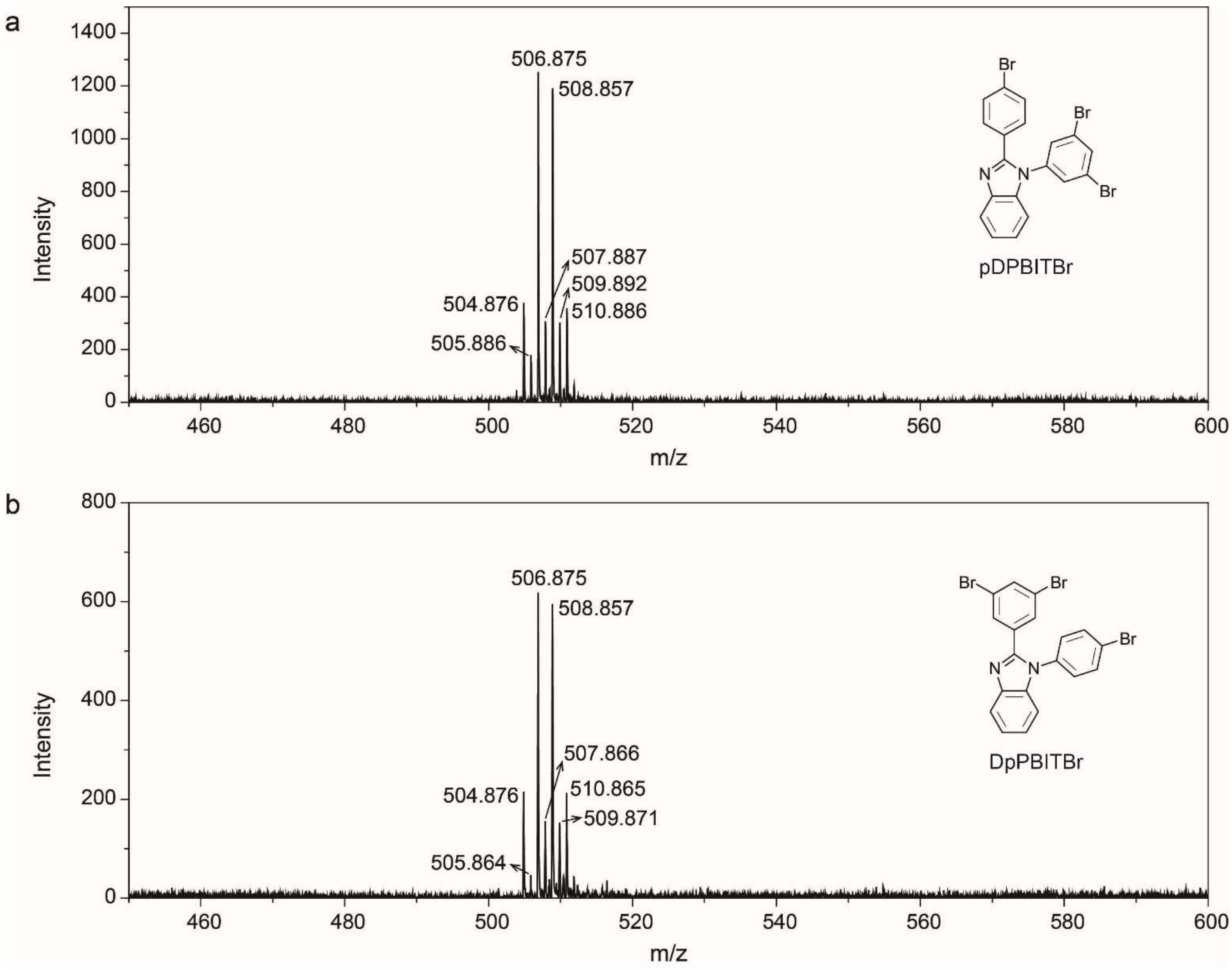

Supplementary Fig. 25 High-resolution mass spectra of pDPBITBr (**a**) and DpPBITBr (**b**).

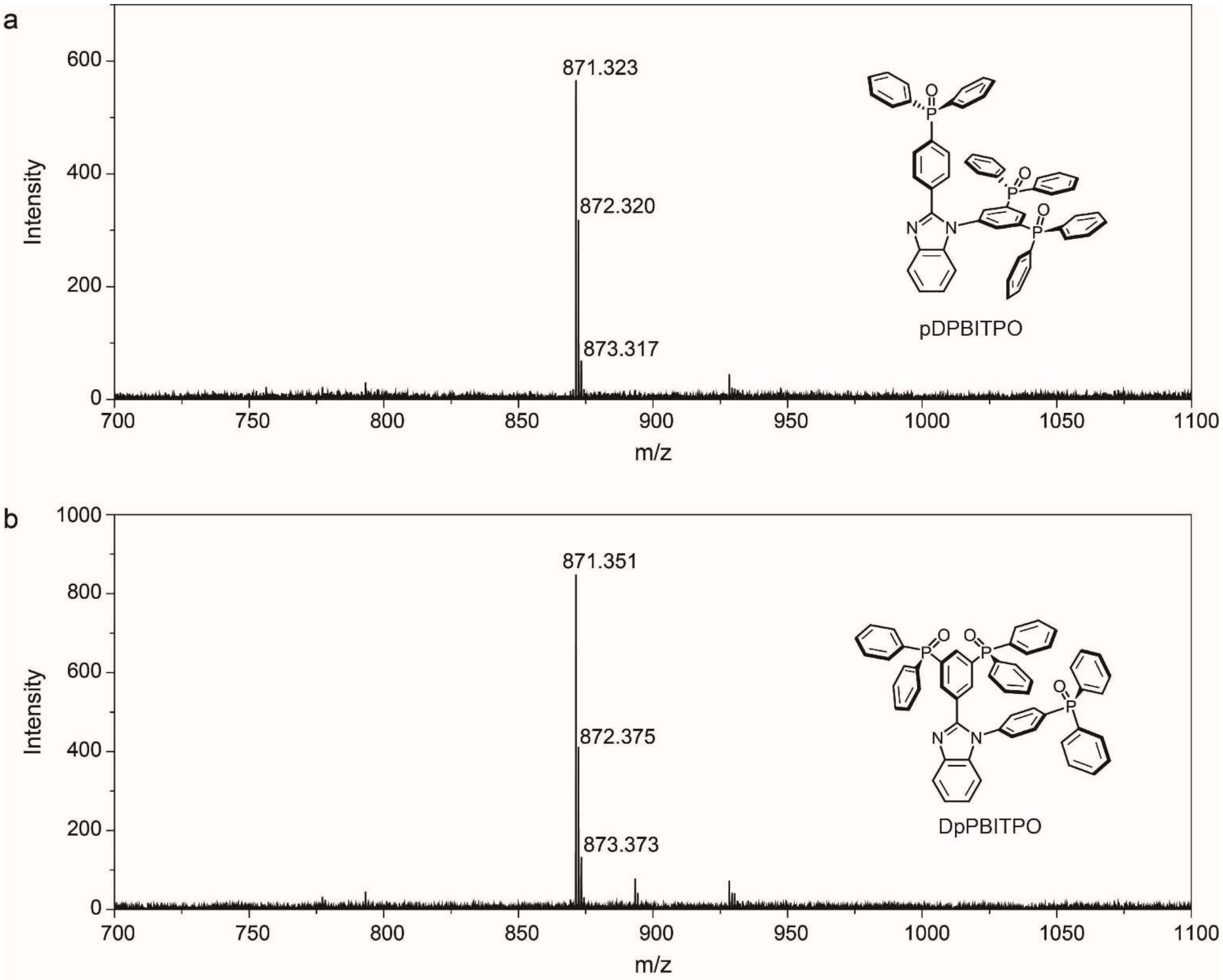

Supplementary Fig. 26 High-resolution mass spectra of pDPBITPO (**a**) and DpPBITPO (**b**).

Supplementary Table 1 Physical properties of *pD*PBITPO and *Dp*PBITPO.

Compound	Absorption (nm)	Emission (nm)	S_1 (eV)	T_1 (eV)	$T_g/T_m/T_d$ (^oC)	HOMO (eV)	LUMO (eV)	μ_e[h] (10^{-4} cm^2 V^{-1} s^{-1})
***pD*PBITPO**	229,283,302[a]	383[a]	3.24[c]	2.48[e]	-/203/504	-6.51[f]	-3.10[f]	0.76
	229,281,309[b]	382[b]	4.41[d]	2.57[d]		-6.20[g]	-1.79[g]	
***Dp*PBITPO**	228,281,306[a]	384[a]	3.23[c]	2.48[e]	-/251/513	-6.51[f]	-3.13[f]	1.33
	228,277,307[b]	376[b]	4.60[d]	2.72[d]		-6.23[g]	-1.83[g]	

[a]In CH_2Cl_2 (10^{-6} mol L^{-1}). [b]In film. [c]Estimated according to the steady-state emission peak wavelength. [d]TD-DFT calculated results. [e]Calculated according to the 0→0 transitions of phosphorescence spectra. [f]Calculated according to the equation HOMO/LUMO = -(4.78 + onset voltage). [g]DFT calculated results; [h]Zero-field electron mobility evaluated with *I-V* characteristics of electron-only devices according to field-dependent space charge-limited current (SCLC) model.

Supplementary Table 2 Physical properties of *m*CP:*pD*PBITPO and *m*CP:*Dp*PBITPO.

Exciplex	Emission (nm)	ϕ_{PL}[e] (%)	τ_{DF}[f] (μs)	S_1 (eV)	T_1 (eV)	HOMO (eV)	LUMO (eV)	μ[k] (10^{-5} cm^2 V^{-1} s^{-1})
***m*CP:*pD*PBITPO**	415[a]	11[a]	5.7[a]	2.99[g]	2.98[i]	-5.404[j]	-1.513[j]	3.37
	480[b]	33[b]	1.2[b]	3.41[h]	2.98[h]			0.49
	566[c]	61[c]	2.7[c]					
	471, 555[d]	92[d]	0.62, 1.1/0.74[d]					
***m*CP:*Dp*PBITPO**	412[a]	14[a]	5.5[a]	3.01[g]	3.00[i]	-5.405[j]	-1.521[j]	4.77
	480[b]	35[b]	1.1[b]	3.45[h]	3.02[h]			3.35
	565[c]	68[c]	2.9[c]					
	474, 551[d]	97[d]	0.39, 0.69/0.37[d]					

[a]Neat films. [b]DMAC-DPS-doped films (30 wt.%). [c]4CzTPNBu-doped films (5 wt.%). [d]DMAC-DPS (30 wt.%) and 4CzTPNBu (0.5 wt.%) codoped films. [e]Absolute PLQY measured with an integral sphere. [f]Delayed fluorescence lifetime. [g]Estimated with the peak wavelength of steady-state PL. [h]TD-DFT calculated results. [i]Estimated according to the 0→0 transitions of phosphorescence after 100 μs with time-resolved technology. [j]DFT calculated results. [k]Zero-field electron and hole mobility evaluated with *I-V* characteristics of single-carrier transporting devices according to field-dependent space charge-limited current (SCLC) model.

Supplementary Table 3 EL performance of representative exciplex-hosted and/or TADF white OLEDs.

Device structure	V^a (V)	L_{Max} (cd m^{-2})	η^b			λ^c (nm) CIE (x, y)	Ref.
			η_{CE} (cd A^{-1})	η_{PE} (lm W^{-1})	η_{EQE} (%)		
PEDOT:PSS|NPB|TCTA:Ir(MDQ)$_2$(acac)|TCTA:Ir(ppy)$_2$(acac)|TCTA:TmPyPb:4P-NPD|Cs_2CO_3	3.1, 3.6, 3.9	~50000	45.2, -, 40.5	41.7, -, 34.3	19.0, -, 17.0	430, 520, 600 (0.43, 0.43)	*Adv. Mater.* **2014**, *26*, 1617
HATCN|TrisPCz|mCBP:4CzPN|mCBP:4CzPN:4CzTPN-Ph|PPT:3CzTRZ|PPT|LiF	3.6, 5.4, 7.8	~4000	45.6, 32.4, 20.6	34.1, 18.8, 8.3	17.0, 13.3, 8.5	480, 580 (0.33, 0.41)	*Appl. Phys. Lett.* **2014**, *104*, 233304
TAPC|CDBP|CDBP:PO-T2T:Ir(ppy)$_2$acac:Ir(MDQ)$_2$acac|PO-T2T|LiF	2.5, 3.4, 4.8	<20000	67.0, 53.2, 37.0	84.1, 48.7, 24.2	25.5, 20.2, 14.1	466, 507, 584 (0.40, 0.43)	*Adv. Mater.* **2015**, *27*, 7079
MoO_3|NPB|mCP|SFXSPO:4CzPNPh|SFXSPO:TPXZPO|SFXSPO|Bphen|LiF	3.5, <6.0, <8.0	> 20000	42.3, 38.5, 35.1	38.0, 20.1, 13.7	16.3, 14.8, 13.5	480, 564 (0.42, 0.45)	*Chem. Mater.* **2016**, *28*, 5667-5679
MoO_3|CDBP|CDBP:PO-T2T:2CzPN:AnbCz|PO-T2T|LiF	2.3, 2.7, 4.3	<10000	50.1, -, -	63.0, -, 10.3	19.0, -, 5.6	470, 560 (0.34, 0.44)	*ACS Appl. Mater. Interf.* **2016**, *8*, 32984
TAPC|TCTA|26DCzPPy:Ir(2-phq)$_2$acac|26DCzPPy:PO-T2T| PO-T2T|LiF	2.6, 3.7, 4.9	<20000	35.5, 33.1, 25.9	41.3, , 15.9	19.3, 18.0, 14.1	485, 597, 643 (0.50, 0.38)	*ACS Appl. Mater. Interf.* **2016**, *8*, 26135

MoO_3|TAPC:MoO_3|TAPC|PO-01|*m*CP:B3PYMPM:FIrpic|B3PYMPM|B3PYMPM:Li_2CO_3|Li_2CO_3	2.4, 2.7, 3.3	<30000	64.5, -, 62.8,	75.3, -, 63.1	20.0, -, 19.5	472, 505, 556 (0.42, 0.51)	*ACS Appl. Mater. Interf.* **2016**, *8*, 10093
TAPC|TCTA|*m*CP|*m*CP:B4PyMPM:FIrpic:PO-01|B4PyMPM| Liq	2.5, 2.9, 3.6	<20000	86.3, 75.3, 61.0	105.0, 82.5, 59.5	28.1, 24.0, 21.5	472, 556, 588 (0.40, 0.48)	*Adv. Funct. Mater.* **2017**, *27*, 1701314
NPB|*m*CP|PHCz2BP:Ir(ppy)$_2$acac:Ir(bzq)$_2$dipba:Ir(bt)$_2$dipba|B3PYMPM|LiF	2.7, ~4, ~5.5	25540	-, -, -	48.3, 34.5, 24.1	25.6, 25.3, 25.1	460, 510, 600 (0.41, 0.46)	*Adv. Funct. Mater.* **2018**, *28*, 1707002
HATCN|TCTA:20%HATCN|TCTA|*m*CP|TCTA:Ir(ppy)$_2$acac:Ir(PPQ)$_2$acac|DPEPO:DMAC-DPS|DPEPO|BmPyPB:3% Li_2CO_3|Li_2CO_3	3.2, -, 4.2	~10000	51.0, -, 39.5	51.7, -, 19.5	23.0, -, 17.5	470, 510, 600 (0.44, 0.44)	*Adv. Funct. Mater.* **2016**, *26*, 3306
PEDOT:PSS|TPAC|*m*CP|*m*CP:PO-T2T|PO-T2T|Liq|Al|MoO_3|DTAF|DTAF:PO-T2T|PO-T2T|Liq	～5.0, 8.7, 11.8	50300	27.7, 26.3, 25.0	15.8, 9.5, 6.8	11.6, 11.0, 10.5	475, 563 (0.29, 0.35)	*Sci. Rep.* **2014**, *4*, 5165
HATCN|TAPC|*m*CP|TXOPhCz4:*o,o*'-NPh_2|TmPyPB|LiF	3.5, ~5, ~6	~10000	30.2, 22.5, 18.8	27.1, 14.4, 9.8	12.5, 9.3, 7.8	450, 580 (0.38, 0.40)	*J. Mater. Chem. C* **2018**, *6*, 2951
TAPC|CBP:3,6-2TPA-TXO|CBP|CBP:3,6-2TPA-TX|TmPyPB|LiF	3.4, 6.7, 9.1	-	49.5, 14.9, 8.5	48.6, 6.9, 2.9	20.4, 6.9, 1.3	470, 580 (0.32, 0.40)	*Adv. Funct. Mater.* **2016**, *26*, 6904
NPD|*m*CP:DBP:TTPA|*m*CP|DMACDPS|DPEP	~3, ~3.5, ~4	~10000	-, -, -	-, -, -	12.1, -, -	475, 610, 670	*Adv. Mater.*

O|TPBi|LiF						(0.25, 0.31)	**2015**, *27*, 2019
HATCN|TAPC|CBP:NI-1-PhTPA|CBP:PXZDSO2|CBP:NI-1-PhTPA|TmPyPB|LiF	3.6, -, -	>10000	43.3, -, 36.1	33.6, -, 17.7	15.8, -, 13.3	440, 560 (0.40, 0.48)	*Adv. Mater.* **2016**, *28*, 4614
PEDOT:PSS|*m*CP|CzAcSF:TBPe|TSPO1|TPBi|LiF	~3, ~4.5, ~5	~2000	32.6, -, 24.8	33.4, -, 16.4	15.2, -, 12.2	470, 490, 550 (0.23, 0.30)	*Adv. Mater.* **2015**, *27*, 4358
MoO_3|NPB|*m*CP|SFXSPO:DMAC-DPS:4CzPNPh|TPBi|LiF	3.9, <5.5, <7.3	~15000	50.5, 48.5, 41.4	40.6, 27.6, 17.9	19.1, 18.3, 15.7	480, 560 (0.32, 0.43)	*Adv. Mater.* **2016**, *28*, 3122
MoO_3|NPB|*m*CP|m2tBCzPO:PO-01|TPBi|LiF	3.3, 5.1, 6.8	18720	60.6, 58.2, 54.5	67.8, 40.7, 29.2	21.3, 20.4, 19.2	484, 552 (0.34, 0.42)	*Adv. Opt. Mater.* **2018**, *6*, 1800020
MoO_3|NPB|mCP|SXSPO:DMAC-DPS:4CzTPNBu|SXSPO|Bphen|LiF	3.1, 4.8, 6.9	22430	56.7, 51.0, 43.5	57.5, 34.0, 20.1	22.1, 20.0, 17.0	468, 548 0.31, 0.39	*Chem. Eng. J.* **2019**, *374*, 471-478
HATCN|TAPC:HATCN|TAPC|CBP:FDQPXZ|DPEPO:DMAC-DPS|DPEPO|BmPyPB:Li_2CO_3|Li_2CO_3	2.6, 2.8, 3.2	< 30000	51.3, 46.5, 31.9	59.6, 52.4, 31.7	20.5, 18.8, 13.0	480, 560 (0.33, 0.41)	*Nat Commun* **2019**, *10*, 2380.
MoO_3|mCP|DBFDPO:TtBCzDFTPPO:4CzTPNBu|DBFDPO|LiF	3.1, 3.8, 5.1	20125	60.6, 55.6, 49.1	61.4, 42.5, 30.3	22.3, 20.4, 18.0	480, 544 (0.28, 0.40)	*Adv. Funct. Mater.* **2020**, *30*, 1908568

MoO_3|mCP|*pt*BCzPO2TPTZ:4CzTPNBu|*p*TPOTZ|LiF	3.1, 5.5, 7.9	37160	52.7, 51.7, 46.2	55.1, 32.4, 18.1	23.6, 23.0, 20.7	480, 560 (0.34, 0.36)	*Adv. Mater.* **2020**, *32*, 1906950
PEDOT:PSS|m-ACSO2|3DMAC-BP|m-ACSO2|TmPyPB|LiF	2.5, 3.5, 5.1	< 5000	51.7, 48.3, -	46.4, 43.5, -	24.2, 23.2, -	494， 616 (0.39, 0.43)	*Adv. Mater. Interfaces* **2020**, *7*, 1901758
MoO_3|*m*CP|*m*CP:*pD*PBITPO:35% DMAC-DPS:0.5% 4CzTPNBu|*pD*PBITPO|LiF	2.9, 4.9, 7.1	36760	90.9, 84.3, 75.6	98.4, 54.0, 34.4	30.6, 28.4, 25.4	476, 564 (0.43, 0.48)	*This work*
MoO_3|*m*CP|*m*CP:*Dp*PBITPO:35% DMAC-DPS:0.2% 4CzTPNBu|*pD*PBITPO|LiF	2.9, 5.0, 9.3	18120	70.1, 66.4, 60.0	74.4, 42.1, 23.7	26.5, 25.1, 22.7	476, 556 (0.31, 0.35)	*This work*
MoO_3|*m*CP|*m*CP:*Dp*PBITPO:35% DMAC-DPS:0.5% 4CzTPNBu|*pD*PBITPO|LiF	2.7, 4.2, 6.7	37000	93.1, 89.8, 84.3	108.2, 68.4, 39.5	32.7, 31.5, 29.6	476, 560 (0.44, 0.47)	*This work*
MoO_3|*m*CP|*m*CP:*pD*PBITPO:35% DMAC-DPS:0.5% 4CzTPNBu|*pD*PBITPO|LiF (10 in^2)	2.7, 4.7, 7.7	2060	71.1, 49.8, 32.0	82.7, 33.2, 13.0	25.0, 17.5, 11.2	480, 556 (0.41, 0.47)	*This work*
MoO_3|*m*CP|*m*CP:*Dp*PBITPO:35% DMAC-DPS:0.5% 4CzTPNBu|*pD*PBITPO|LiF (10 in^2)	2.7, 4.5, 7.7	2130	74.5, 63.4, 48.6	86.6, 48.4, 19.8	26.1, 22.4, 17.0	480, 556 (0.40, 0.46)	*This work*

[a]Operating voltages at 1, 100 and 1000 cd m^{-2}. [b]Efficiencies at the maximum, 100 and 1000 cd m^{-2}. [c]EL emission peak.
